# Differential brain mechanisms during reading human vs. machine translated fiction and news texts

**DOI:** 10.1038/s41598-019-49632-w

**Published:** 2019-09-13

**Authors:** Fa-Hsuan Lin, Yun-Fei Liu, Hsin-Ju Lee, Claire H. C. Chang, Iiro P. Jaaskelainen, Jyh-Neng Yeh, Wen-Jui Kuo

**Affiliations:** 1 Institute of Biomedical Engineering, National Taiwan University, Taipei, Taiwan; 2 Department of Medical Biophysics, University of Toronto, Toronto, Canada; 3 Department of Neuroscience and Biomedical Engineering, Aalto University, Espoo, Finland; 4 Institute of Neuroscience, National Yang-Ming University, Taipei, Taiwan

**Keywords:** Cognitive neuroscience, Language

## Abstract

Few neuroimaigng studies on reading comprehension have been conducted under natural reading settings. In this study, we showed texts presented in a natural way during functional MRI (fMRI) measurements to reveal brain areas sensitive to reading comprehension. Specifically, this paradigm independently manipulated two holistic features of article style: text genre and translation style, a qualitative index of how typical word choices and arrangements are made in daily use of the language. Specifically, articles from *The New York Times* (news) and *Reader*’*s Digest* (fiction) translated from English to Mandarin Chinese either by human experts or machine (*Google Translate*) were used to investigate the correlation of brain activity across participants during article reading. We found that bi-hemispheric visual cortex, precuneus, and occipito-parietal junction show significantly correlated hemodynamics across participants regardless of translation style and article genre. Compared to machine translation, reading human expert translation elicited more reliable fMRI signals across participants at precuneus, potentially because narrative representations and contents can be coherently presented over tens of seconds. We also found significantly stronger inter-subject correlated fMRI signals at temporal poles and fusiform gyri in fiction reading than in news reading. This may be attributed to more stable empathy processing across participants in fiction reading. The degree of stability of brain responses across subjects at extra-linguistic areas was found correlated with subjective rating on the text fluency. The functional connectivity between these areas was modulated by text genre and translation style. Taken together, our imaging results suggested stable and selective neural substrates associated with comprehending holistic features of written narratives.

## Introduction

Reading is to understand the meaning encoded in an article composed of a hierarchical structure of paragraphs, sentences, and words. Although there have been extensive studies examining the neural basis of reading words and sentences, how the brain activity is modulated by holistic features of an article, such as its content and style, during reading comprehension is still poorly understood.

Categorizing an article into a fiction or non-fiction defines one of its holistic features. Previously, it was suggested that the neural substrates for social cognition and fiction comprehension can overlap^1^. As humans are social animals and tend to be perpetually prepared for social interaction^2–4^, our brain has been evolved to support social cognitive functions, including inferring others’ mental states and their perspective taking, in order to better predict up-coming events^5^. Accordingly, fiction reading can be considered as a mental exercise to simulate the social experience^1,6,7^, because it entails readers to get into others’ thoughts and feelings and, therefore, gives a vivid simulation of the reality. This hypothesis on the linkage between social cognition and fiction comprehension can be specifically tested by reading materials with much reduced inter-person social relationships, such as news.

The other holistic feature of an article is its word choices and arrangements. While the same information can be carried by different word choices and arrangements, however, distinct representations directly modulate the effectiveness of information transmission during reading. One concrete example is reading versions of translated texts from the same article of another language. The contrast between comprehending different translated texts is particularly prominent between reading the translation from a human professional and from machine. It is commonly believed that the closer a machine translation to a professional human translation, the better. However, the difference in the neural substrate between reading human and machine translation remains unknown.

Recently, it has been advocated to study neural and cognitive bases of comprehending literary work in a neurocognitive approach^6,8,9^. However, probing the neural processes related to two above-mentioned holistic features of an article is difficult. It is potentially because of the lack of a proper method to measure the associated effects, which are likely accumulated over the reading time^8,9^. Instead of using the well-controlled elementary components to understand how brain accomplishes complicated tasks, there have been studies measuring stable brain responses across participants elicited by complex and naturalistic stimuli, including movies, TV shows, and musical pieces, in order to unveil how brain works^10–12^ (for a review, see^13^). In language studies, this approach has been used to better understand speech comprehension by story listening and telling paradigms^14–19^. However, to the best of our knowledge, neural substrates underpinning reading comprehension has not been explored by presenting reading materials in a naturalistic fashion^20–23^.

In this study, we attempted to reveal neural correlates of reading comprehension by presenting articles using a text scrolling technique to mimic naturalistic reading. We particularly interrogated how the degree of regional synchronized brain responses across participants can be modulated by literary genre and translation quality. To this end, we presented texts with news (*The New York Times*) and fictional contents (*Reader*’*s Digest*) translated from English to Mandarin Chinese by human professionals and a computer program (*Google Translate*) to the participants during fMRI scanning. By doing so, we were allowed to orthogonally compare effects of literary genre and translation quality during naturalistic reading. We also calculated the brain-behavior correlation by correlating the subjective behavioral ratings on the texts with the synchronized brain responses of the two factors.

Previous studies using complex and naturalistic stimuli have revealed brain areas supporting narrative comprehension regardless of linguistic representations^24^ or sensory input modality^25^. Their results suggested that fictional comprehension brain areas are distributed across the whole brain, including parietal and temporal cortices of the two hemispheres. In this study, since translation versions and literary genre are two holistic features of a text, functional areas associated with these two factors were expected to be outside linguistic areas underpinning word and lexical processing. Extra-linguistic areas integrating information across sentences and paragraphs were more likely to show stable responses and to correlate with subjective ratings on translation quality. Lastly, we expected that reading fictional texts would involve more affective and empathy processing, as suggested by previous behavioral measurements^26^. These hypotheses were tested by calculating stable fMRI hemodynamics across participants at each brain area separately^27^ and between brain areas^28^.

## Method

### Participants and ethical permission

The experiment was run in accordance with the guidelines of the Helsinki Declaration, and ethics approval was obtained from the Institutional Review Board of National Taiwan University Hospital. There were 24 right-handed, native Mandarin Chinese-speaking, healthy young adults with normal or corrected-to-normal vision (mean age = 25.3 years, age range = 19–38 years, 14 females) participating in the fMRI experiment. The education levels of all participants considered of a high school or higher degree. None of them had a history of neurological or psychological disorders. Written informed consent was obtained before experiment. Data of three participants were discarded from analysis because of incompletion of data collection and stimulus presentation software problems.

### Experimental materials and procedure

Four fictional articles in Chinese were adopted from the Chinese edition (Taiwan English Press, Taipei, Taiwan) of *Reader*’*s Digest* (Reader’s Digest Association, New York City, New York, USA). Four news articles in Chinese (United Daily News Group, Taipei, Taiwan) were adopted from the international weekly edition of *The New York Times* (The New York Times Company, New York City, New York, USA). In this study, FH and NH were used to denote human-translated fictional and news articles, respectively. All FH articles were first-person narrated. NH articles described either scientific discoveries or government policy. Articles were chosen with the consideration to match the character count between FH and NH article pairs. The original English texts of 4 fictional and news articles were also translated by Google Translate (https://translate.google.com.tw/). These machine-translated articles were referred to as FM and NM, respectively. All FM and NM articles were further slightly manually edited: First, functional words were paraphrased and redundant characters were added or removed such that the character count between an FH and an FM article (from the same original English text) matched to each other. The same edit was applied to each pair of NH and NM articles. After editing, the difference in the character count within the four types of articles (*i*.*e*., FH, FM, NH, and NM) was less than 3%. Second, translations of proper nouns were edited to ensure consistency between articles. Third, obvious semantic errors were corrected. Note that these edits did not alter the text quality and genre derived from the original machine translation. Taken together, sixteen articles were prepared with four sets of two-(fiction *vs*. news)-by-two (human *vs*. machine) design.

With controlled total character counts, we also analyzed the number of content words in each article after excluding pronouns. The word frequency count of content words in all articles were calculated by looking up the Academia Sinica Balanced Corpus of Modern Chinese (http://asbc.iis.sinica.edu.tw/, Academia Sinica, Taipei, Taiwan). Then, content words were further sorted into four categories based on word occurrence in the corpus: very-low-frequency (<30), low-frequency (30–300), high-frequency (300–3000), very-high-frequency (>3000) among 17,554,089 total occurrences. There were more content words in news articles (344) than in fictional articles (269; *p* < 0.05). News articles also contained more low-frequency and very-low-frequency words than fictional articles. The differences in high- and very-high-frequency word counts between news and fictional articles were not significant. There was no significant difference in the number of content word between human- and machine-translated articles.

Articles were visually presented on a rear projection screen mounted at the subject’s head vertex end of the MRI bore. Texts were shown in a vertical scrolling fashion: a gradient mask was applied so that only four to five lines of texts were visible within a 10° viewing in order to control vertical eye movements. Participants viewed stimuli via a mirror affixed to the MRI head coil array. Stimulus presentation was controlled by using E-Prime 2.0 software (Psychology Software Tools, Pittsburgh, PA).

The scrolling speed was determined by a speed pilot test in which eight right-handed, native Mandarin Chinese-speaking participants (mean age = 23.5 years, age range = 20–29 years, 4 females) were recruited for testing. Each participant was asked to read four articles (FH, FM, NH, and NM) and inform the experimenter when they finished reading one article sequentially. After reading an article, each participant was asked to answer two multiple-choice questions to ensure he/she understood the content of the article. Based on the pilot test, the scrolling speed of each article presentation was set to show a 1300-character article in about 180 s. All participants answered multiple-choice questions by the end of the article presentation, indicating that they understood articles despite the genre differences.

In the fMRI experiment, participants were instructed to read eight articles: 2 articles for FH, FM, NH, and NM, respectively. Prior to the presentation of an article, a cross for visual fixation was shown for 4 s to inform the participant. Then the title of the article was shown for 5 s to facilitate comprehension^29^. Titles for articles of different translation were identical. Another visual fixation cross was presented to indicate the end of an article presentation. Each participant was asked to answer a multiple-choice question using button pressing to ensure he/she understood the article. Afterward, the experimenter asked the participant to rate the fluency of the article in a scale from 1 (the least fluent) to 10 (the most fluent). After reading the first four articles, participants were asked to rest for five minutes. Anatomical MRI was acquired during the resting period. The order of eight articles was arranged such that no two articles of the same combination of literary and translation style were presented consecutively. No article of two translations from the same original text were presented to the same participant.

### MRI data acquisition

Participants were scanned in a 3T MRI scanner (Tim Trio, Siemens, Erlangen, Germany) using a 32-channel head coil array. Images were acquired using a ${T}_{2}^{\ast }$-weighted echo-planar imaging (EPI) pulse sequence (TR = 2000 ms; TE = 30 ms; flip angle = 90°). Each volume had 33 slices, each of which was 3.5 mm thick with 10% gap. The in-plane resolution was 3.4 × 3.4 mm^2^ with a 220 × 220 mm^2^ field-of-view (FOV). Anatomical images were acquired using a *T*_1_-weighted magnetization-prepared rapid-acquisition gradient echo (MPRAGE) pulse sequence (TR = 2530 ms; TE = 3.03 ms; flip angle = 7°; 1 mm^3^ resolution). Foam padding was used to support the subject’s head and to minimize head movement. Subjects were also asked to avoid head movements when reading texts and answering questions.

### Data analysis

Reconstruction of both cortical surfaces and fMRI data preprocessing were done with Freesurfer (http://surfer.nmr.mgh.harvard.edu/) and MATLAB (MATLAB 7.13, The MathWorks, Inc., Natick, Massachusetts, United States). Preprocessing of fMRI data included intra-session 3D motion correction, slice-timing correction, linear and sinusoidal confounder removal, and spatial smoothing (using a Gaussian smooth kernel with full-width-half-maximum of 10 mm). EPI time series from all participants were transformed to a standard template using a spherical coordinate system to facilitate subsequent inter-subject correlation analysis.

### Inter-subject correlation calculation

We calculated inter-subject correlation (ISC) maps for each condition. In our experiment, the combination of literary style (2: F or N), translation type (2: H or M), and indices (4: #1, #2, #3, #4) yielded 16 conditions. Here we used a three-letter index to specify the article presented to the participant. For example, FH1 represented the first time showing of the human translated fictional article to our participants. ISC quantifies the correlation between the time courses of BOLD activity at the same cortical area between two participants when they read the same article.

Since the lengths of all articles were not identical, we truncated the fMRI time series to get the first 94 time points for subsequent analysis. A correlation map between pairs of participants across all cortical locations was calculated for each article. Because each article was read by 10 participants, we obtained $$(\begin{array}{c}10\\ 2\end{array})=45$$ correlation maps for each article. The ISC values at each cortical location in each map were first transformed to *Z*-statistics using the Fisher’s Z transformation^30^ and then analyzed using General Linear Model (GLM) to reveal effects related to literary and translation styles. We created four regressors to respectively probe the average effect of a combination of literary and translation style in the GLM analysis. Accordingly, the dimension of the design matrix in GLM was 720 × 4. For example, in the column representing the effect related to reading FM articles, only entries corresponding to ISC values computed from reading a FM article was set to 1, while other entries were set to 0.

Based on the GLM analysis, we found statistically significant ISC values for reading different literary (F or N) and translation style (H or M). For example, significance ISC values in reading M articles were calculated by using a contrast vector [0.5 0.5 0 0]^T^ for the design matrix with the first two regressors representing the effect of reading news and fiction articles translated by machines. Similarly, we statistically compared the ISC values between reading different literary (F *vs*. N) and translation style (H *vs*. M). The interaction between literary and translation style was also investigated. The reported statistical significance was corrected for multiple comparison by using an FDR-adjusted p value < 0.05^31^.

### Inter-subject functional connectivity calculation

We further analyzed the correlated brain activity between areas using inter-subject functional connectivity (ISFC) analysis^28^. Specifically, brain areas showing significantly different ISC values between reading of different literary (F *vs*. N) and translation style (H *vs*. M) were first chosen as seed regions-of-interest (ROI’s). All time series within a seed ROI were first averaged from one chosen participant. This seed ROI time series was correlated to the average time series across all other participants over the whole brain. These correlation coefficients were Fisher Z-transformed to generate an ISFC map. This procedure was repeated until all participants provided the seed ROI time series for separate ISFC maps calculations. ISFC maps were further analyzed using GLM to reveal brain networks showing different connectivity to the chosen seed ROI across literary and translation styles. The ISFC analyses were repeated over all chosen seed ROI’s.

## Results

### Behavioral results

Participants subjectively reported that reading FH articles required the least effort. Reading FH articles was faster than reading others (*t*-test; *p* < 0.0005). Reading human-translated articles was quicker than reading machine-translated articles (*t*-test; *p* < 0.005).

In the fMRI experiment, participants correctly answered 94% of the multiple-choice questions about the content of articles. Based on the *t*-test, the 94% correct answer rejected this null hypothesis (*p* < 0.001). In average, machine-translated articles received a fluency rating of 5.28, whereas human translated articles were rated 7.33. The scores were significantly different (*p* < 0.0001). The difference between the average ratings of fictional (6.57) and news (6.04) articles did not differ significantly.

### fMRI results

**Figure 1:**
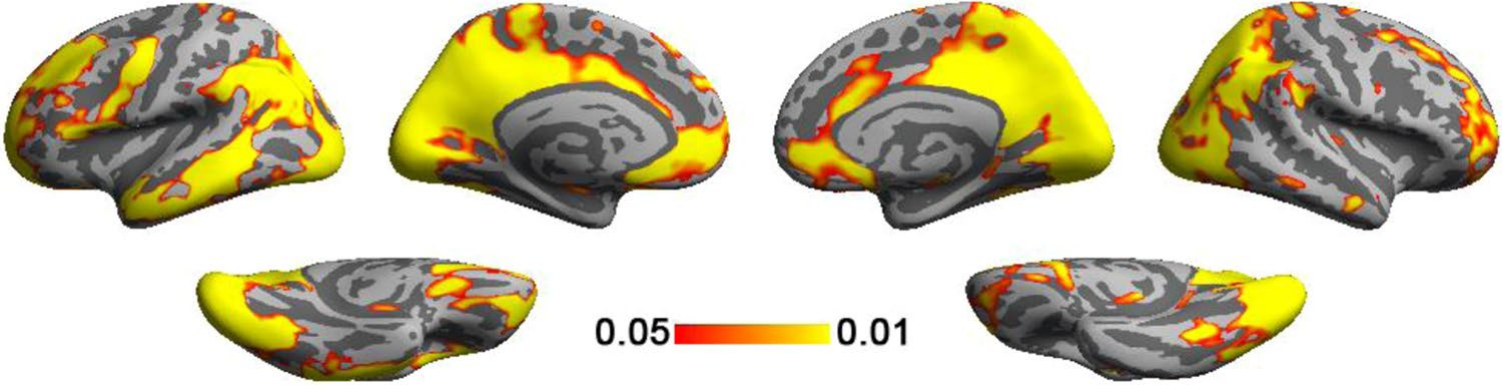
Grand average of significant (*p* < 0.05 after FDR correction) ISC maps.

The grand average of significant ISC across article genres and translations was shown in Fig. 1. Stable BOLD signal time courses were found at visual cortex, posterior cingulate cortex/precuneus, medial prefrontal cortex, anterior lateral prefrontal cortex, and parietal lobes of both hemispheres. Left middle and inferior temporal gyri, as well as left inferior frontal gyrus, had significantly correlated BOLD signal across subjects.

**Figure 2:**
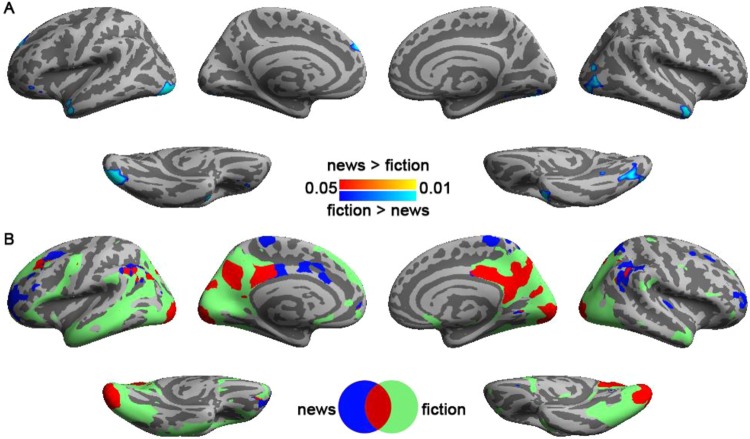
Comparison (**A**) and conjunction (**B**) analyses on the inter-subject correlated BOLD signals when participants read news and fiction articles. Both analyses used an FDR-adjusted p value < 0.05.

Figure 2 shows the distributions of significant ISC when participants read news and fiction articles. We found that reading fiction articles elicited larger ISCs than reading news articles in occipital lobe, parietal lobe, left inferior/medial temporal gyri, and right temporal pole. The areas showing significant ISC regardless of article genre included bi-hemisphere visual cortex, precuneus, and intraperietal sulci (BA 39). Reading fiction articles specifically caused significant ISC in left dorsal lateral prefrontal cortex (BA 9), left inferior frontal gyrys (BA’s 44 and 45), and right temporal pole (BA 38), while reading news articles specifically caused significant ISC in bi-hemispheric rostral prefrontal cortex (BA 10). Statistical comparison on ISC between reading these two kinds of articles shows that only reading fiction articles had stronger ISC than reading news articles at the lateral occipital sulcus (BA’s 18 and 19), bi-hemispheric temporal pole (BA 38), and left dorsal lateral prefrontal cortex (BA 9). Coordinates and anatomical labels of this comparison are listed in Table 1.

**Table 1:** MNI coordinates of significant clusters (corrected p < 0.05; cluster area >100 mm^2^) of ISC comparisons.

Effect	Max Z	Area (mm^2^)	X	Y	Z	Anatomical label
Human > Machine	3.456	449.73	16.2	−71.1	46.4	Superior parietal
	3.284	133.86	11.8	−79.6	35.4	cuneus
Fiction > News	−3.408	282.59	−6.9	55.3	30.9	Superior frontal
	−3.407	420.22	−23.8	−84.3	−8.4	fusiform
	−3.407	174.49	−52.3	12	−11.5	Superior temporal
	−3.4	119.97	−51.5	0.7	−25.3	Middle temporal
	−3.408	300.26	44	6.4	−29.4	Middle temporal
	−3.408	1018.04	44	−77.1	7.1	Lateral occipital
	−3.406	117.86	36.6	−79.3	13.8	Lateral occipital
News (Human - Machine) > Fiction (Human - Machine)	3.386	1512.72	−5.6	−12.4	54.1	Paracentral
	3.385	679.27	−28.9	2.4	−35.2	fusiform
	3.38	699.22	−64.8	−22.2	3.2	Superior temporal
	3.377	163.72	−55.3	4.5	4.8	Precentral
	3.316	620.47	−44.3	−36.9	12.2	Superior temporal
	3.386	1098.87	8	−49.2	61.1	Precuneus
	3.382	164.49	36.5	−85.4	−8.3	Lateral occipital
	3.143	214.64	40.4	−5.8	−34.7	Inferior temporal

**Figure 3:**
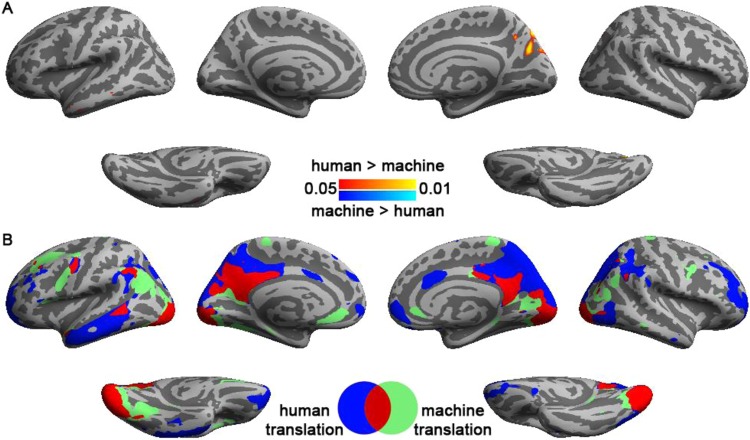
Comparison (**A**) and conjunction (**B**) analyses on the inter-subject correlated BOLD signals when subject read human- and machine-translated articles. Both analyses used an FDR-adjusted p value < 0.05.

Figure 3 shows the distributions of significant ISC when participants read human- and machine-translated articles. Reading machine translation specifically elicited significant ISC at left intra-parietal sulcus (BA 39), left inferior frontal gyrys (BA’s 44 and 45), and left dorsal lateral prefrontal cortex (BA 9), while reading human translation specifically elicited significant ISC at left inferior/medial temporal gyri, intra-parietal sulcus (BA 39), and frontal pole (BA 10). Bi-hemispheric visual cortex and precuneus were activated for both translation styles. Smaller clustered ISC’s were also observed around bihemispheric intra-parietal sulcus (BA 39), left inferior frontal gyrys (BA’s 44 and 45), left posterior part of the superior temporal sulcus, and ventral premotor cortex (BA 6). Between human and machine translations, only left precuneus showed statistically higher ISC in reading human translation than reading machine translation articles. Coordinates and anatomical labels of this comparison were listed in Table 1.

**Figure 4:**
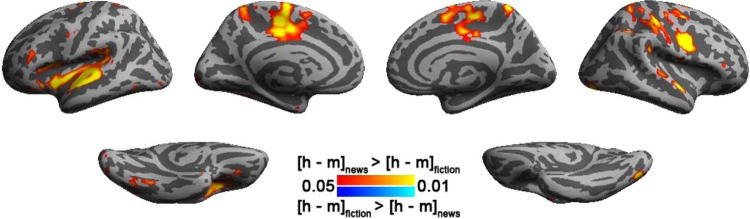
The difference of inter-subject correlated BOLD signals between reading human and machine translations depends on the article genre. This analysis used an FDR-adjusted p value < 0.05.

To investigate if the ISC difference between translations depended on the ISC difference between article genres, we calculated the interaction effect in the analysis. Figure 4 shows that the ISC difference between translations was statistically larger in reading news than reading fiction articles at bi-hemispheric medial parts of the motor area, left insula, left superior temporal gyrus, and right inferior frontal sulcus. Table 1 lists coordinates and anatomical labels of this interaction effect.

**Figure 5:**
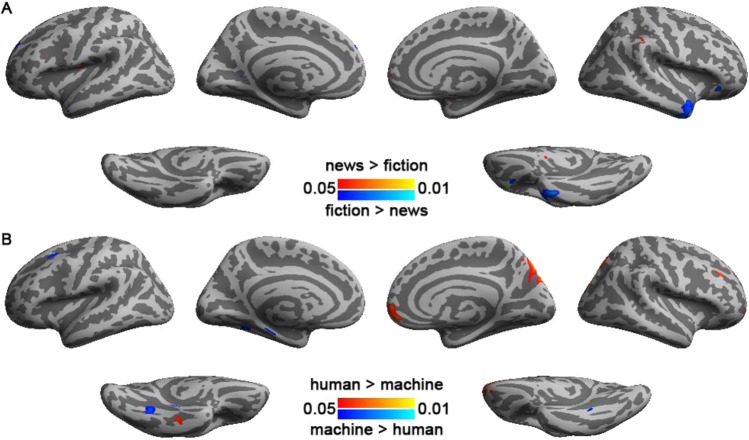
Difference on correlating inter-subject correlated BOLD signals to subjective rating on the text fluency between reading news and fiction (**A**) and between reading human and machine translations. (**B**) This analysis used an FDR-adjusted p value < 0.05.

We further correlated between the ISC values and subjective ratings on text fluency to test if different ways of translation would be associated with synchronized brain activity across participants. Specifically, at each brain location, we used GLM to calculate the correlation between ratings across participants and averaged ISC across all participants (the sum over rows of the inter-subject ISC matrix except the diagonal terms). The comparison between conditions of these correlations was shown in Fig. 5A. Significantly higher correlation between subjective rating on the text fluency and BOLD signal ISC was found at right temporal pole when reading news articles than reading fictions. This location was also found significantly higher ISC when reading fiction (Fig. 2). Reading human translated articles led to higher correlations at right precuneus and right medial frontal lobes than reading machine translated article (Fig. 5B). Reading machine translated articles led to higher correlations at left fusiform gyurs and the left caudal medial frontal lobe than reading human translated article. Table 2 lists coordinates and anatomical labels of the comparison on the correlation between subjective rating and ISC between conditions.

**Table 2:** MNI coordinates of significant clusters (corrected p < 0.05; cluster area >100 mm^2^) of the comparisons on the correlations between subjective rating on text fluency and ISC.

Effect	Max Z	Area (mm^2^)	X	Y	Z	Anatomical label
Human > Machine	4.106	625.84	18.1	−71.3	45.4	Superiorparietal
	3.929	670.35	12.7	63.8	−3.4	Frontal pole
	3.88	362.51	11.8	−83.5	37.6	Cuneus
Machine > Human	−3.606	156.5	−30.2	−54.2	−10.7	Fusiform
	−3.474	101.98	−24.3	13.8	42.5	Caudal middle frontal
Fiction > News	−4.143	881.13	47.3	−4.7	−33.2	Inferior temporal
	−3.581	130.68	−9.9	56.6	28.4	Superior frontal

**Figure 6:**
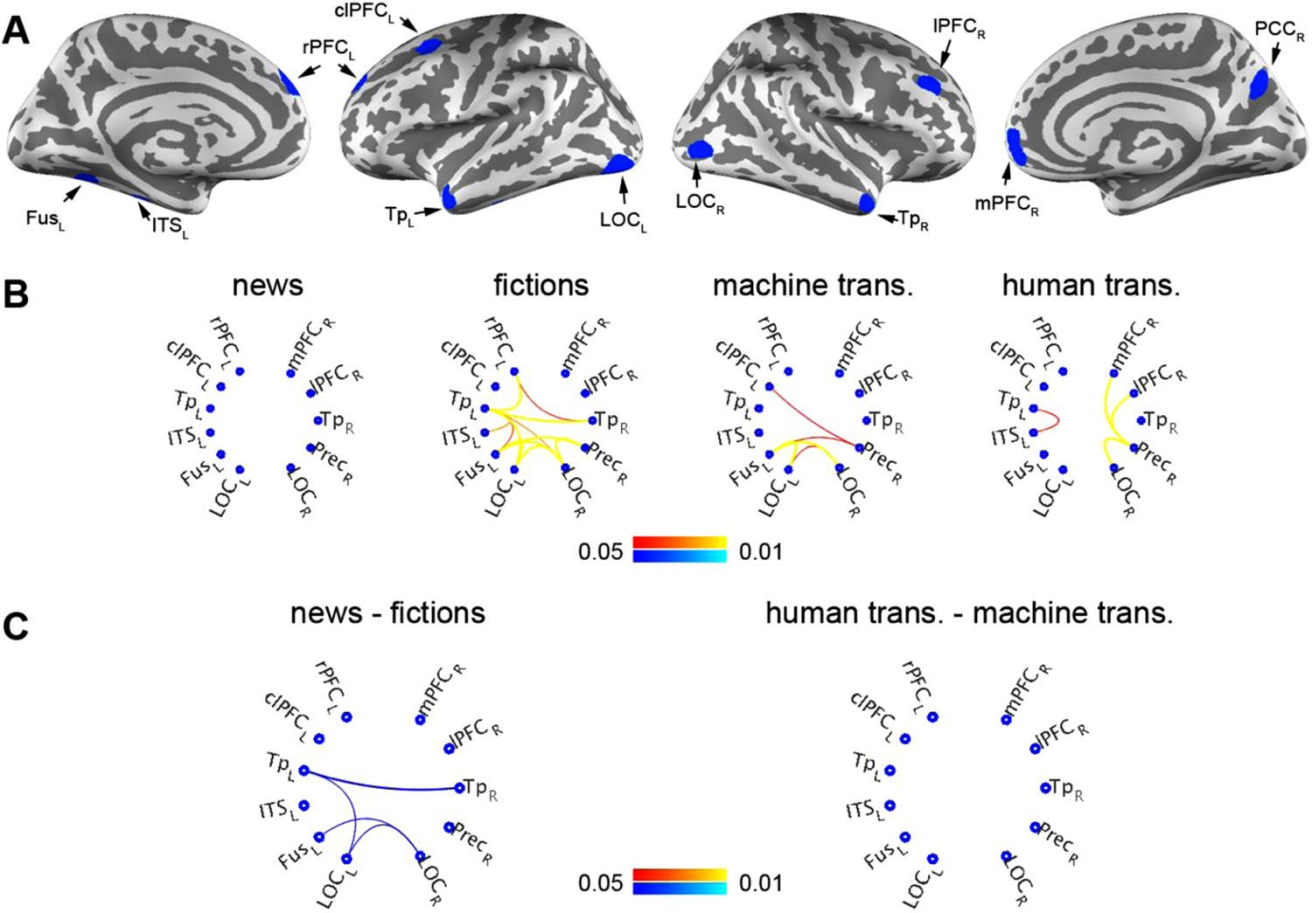
Inter-subject functional connectivity (ISFC) analyses during text reading. (**A**) Brain areas showing significant ISC differences as well as correlation between ISC and subjective ratings on the text fluency were included in the ISFC analyses. rPFC: rostal prefrontal cortex; clPFC: caudal-lateral prefrontal cortex; Tp: temporal pole; ITS: inferior temporal sulcus; Fus: fusiform gyrus; LOC: lateral-occipital cortex; Prec: precuneus; lPFC: lateral prefrontal corex; mPFC: medial prefrontal cortex. The capital letter by the end of each region indicates left (L) or right (R) hemisphere. (**B**) Significant ISFC of news reading, fiction reading, reading texts translated by machine, and translated by human. (**C**) Significant ISFC differences between news and fiction reading as well as between reading texts translated by human and machine. This analysis used an FDR-adjusted p value < 0.05.

To further elucidate how different brain areas transmit and integrate information during reading, we analyzed the inter-subject functional connectivity (ISFC) between 11 seed ROIs showing significant ISC differences between text genres (Fig. 2), between translations (Fig. 3), and significant ISC-subjective rating on text fluency relationship differences between text genres as well as between translations (Fig. 5). These areas were rostral prefrontal cortex (rPFC_L), caudal-lateral prefrontal cortex (clPFC_L), temporal pole (Tp_L), inferior temporal sulcus (ITS_L), fusiform gyrus (Fus_L), and lateral occipital cortex (LOC_L) in the left hemisphere and medial prefrontal cortex (mPFC_R), lateral prefrontal cortex (lPFC_R), temporal pole (Tp_R), precuneus (Prec_R), and lateral occipital cortex (LOC_R) in the right hemisphere (Fig. 6A). Using GLM to separate ISFC into categories of text genres and translations, we found that reading news involves more correlated BOLD signals between hemispheres, particularly in temporal, parietal, and occipital lobes (Fig. 6B,C). Significant ISFC between left hemisphere and occipital as well as parietal lobes were found during reading texts translated by machine, while reading texts translated by human was associated with significant ISFC within the hemisphere (Fig. 6B). No significant ISFC difference was found between reading texts translated by machine and human (Fig. 6C).

## Discussion

In this study, we used the BOLD signal time courses recorded when participants read articles in a naturalistic manner for a few minutes to study the neural substrates underpinning reading comprehension. Specifically, we used this approach to study how BOLD signals are modulated by translation style, which was tested by presenting articles translated from English to Mandarin Chinese by either human or machine, and article genre, which included first-person narrative fictions and news reports. Our analyses showed that bi-hemispheric visual cortex (close to primary visual cortex), precuneus, and occipito-parietal junction show significantly correlated BOLD dynamics across participants regardless of translation style and text genre. We also found that translation style and text genre can modulate the correlation of regional BOLD dynamics across participants (Figs 3, 4 and 5). There were article genre- and translation-dependent brain areas. Statistically more significant inter-subject correlated BOLD signals were found during reading fiction (Fig. 2) and human-translation articles (Fig. 3). Areas showing significant ISC were found with significant correlation to subjective rating on text fluency (Fig. 5). Analyses on the functional connectivity between participants during text reading revealed that reading fictions involves more correlated brain activity across hemispheres between parietal and occipital lobes than reading news (Fig. 6). While reading texts translated by either machine or human elicited concerted brain activity across or within hemispheres, respectively, no significant ISC and ISFC was found between reading texts of different translations (Fig. 6). Taken together, our experiment revealed that reading texts across sentences and paragraphs caused stable brain responses at extra-linguistic areas. The degree of brain response stability at these extra-linguistic areas was found correlated with subjective rating on text fluency.

Investigating brain areas subserving naturalistic text reading has been previously reported in reading English^25^. Comparing to the study, we found that reading both English and Chinese texts shows stable BOLD signals at Wernicke’s and Broca’s areas. However, reading Chinese texts shows less stable BOLD signals at right inferior frontal gyrus and right medial prefrontal cortex. In our study, we also found stable hemodynamic responses elicited across participants at bi-hemispheric visual cortex, bi-hemispheric precuneus, and left intra-parietal sulcus (Fig. 1). While these areas were largely reported in the previous study^25^, stable BOLD responses at bi-hemispheric frontal lobe and IPS were not as diffusive as those in a previous study^25^, presumably due to i) the difference between using English and Chinese articles and the difference in syntactic and/or phonological processing, or ii) the way of presenting visual texts. Specifically, texts were visually shown in a rapid serial presentation at the speed matched to the timing of the audio presentation in spoken languages^25^, while we presented texts by vertically scrolling the text.

Stable brain activity during story comprehension using different languages (Russian and English) has been previously investigated using naturalistic auditory stimuli^24^. It was observed that the distributions of similar brain activity across listeners were similar regardless of the chosen language to convey the information. Here, we further demonstrate that reading articles of the same language (Chinese) elicited similar brain activity in parietal, temporal, and frontal lobes regardless of text genre and translation style. Such results may not be surprising since the representations of information in our experiment were more homogeneous than those in the previous study using different languages^24^ or sensory modality^25^. However, we still find that text genre and translation style can selectively modulate the stability of BOLD signal within regions (Figs 3 and 4) as well as between regions (Fig. 6) during article reading. These regionally stable BOLD signals in turn are closely related to the subjective rating on the degree of text fluency (Fig. 5).

In our experiment, we visually presented texts with manipulated text genre and translation style. We considered these finer manipulations may demonstrate better brain activity modulation effects at higher processing hierarchy than an experiment using different languages, where audio features are readily different in the primary sensory level^25^. While we found the stability of BOLD signals at temporal poles (Fig. 2) and right precuneus (Fig. 3) were indeed modulated by text genre and translation style respectively, we also found that text genre affected the stability of BOLD signal at the visual cortex (Fig. 2).

Reading machine-translated texts was hypothesized to involve more top-down processes to facilitate text comprehension. This hypothesis was partially supported by our data (Fig. 3) that although ISCs in the left parietal and frontal regions [BA 39 and 44/45] were activated in reading both human and machine translations, the activated areas spread wider for the machine translations. This may be attributed to stronger involvement of the left frontoparietal network as an attentional modulator of the verbal short-term memory when reading machine-translated articles^32^. However, one thing to note that there is research showing that the eyes move during reading reveals that readers’ eye-movements co-vary with various text parameters (Reichle, Rayner, & Pollatsek, 2003). Research also shows, during sentence reading, reading effects revealed by eye-movements co-register well with the effects revealed by evoked electrophysiological responses (Dimigen, Sommer, Hohlfeld, Jacobs, Kliegl, 2011). Therefore, under our naturalistic reading setup that the participants can move their eyes freely for reading, the findings of ISC differences might suggest different eye-movement patterns involved for reading as well.

On the other hand, reading human translations showed stronger ISC than machine translations in precunes. The role of precuneus in reading comprehension has been previously reported by a study revealing a network of areas, including prefrontal cortex, precuneus, posterior cingulate cortex, and angular gyrus, when contrasting between narrative and sentence comprehension^33^. Precuneus belongs to one cardinal area of the default-mode network, which has been suggested to support self-referential processing and to generate coherent mental representations^34^. In a reading comprehension study, it was shown that precuneus was more active in reading with self-explaining strategy than paraphrasing, potentially due to its role in episodic and semantic memory retrieval^35^. Importantly, a recent study probing the size of temporal receptive window^36,37^ using a real life story listening paradigm also revealed that precuneus was only reliably responding across listeners when story was coherently presented over long time scales (+/−30 s)^37^. Corroborating these findings, our results also suggested that human translation, which was an operationally defined index of lexical representation common in daily life, can elicit more coherent hemodynamics at high-order comprehension areas across readers (Fig. 5). Furthermore, more significant ISFC was found with right precuneus (Fig. 6). In fact, this matches the longer temporal receptive field of precuneus found previously^37^, as texts of higher fluency are represented beyond single words or sentences. Instead, a good style in narratives, as represented by human expert translations, can take tens of seconds to be appreciated across sentences and paragraphs.

In the comparison between fiction and news reading, we found ISC significantly differed at lateral inferior occipital lobe and temporal poles. Fiction reading has been suggested to be associated with better performance on tests of cognition, empathy, and Theory of Mind^26^. Thus areas associated with empathy and Theory of Mind are expected to be more stably activated in fiction reading. It was found that the temporal pole activity and the degree of understanding others’ mental state are correlated^38^. Theory of Mind and empathy processing both suggested that temporal poles involved in understanding other’s mental state without and with emotional processing^39^. A recent stroke study revealed that damage to the temporal lobe impairs affective empathy^40^. Taken together, the higher ISC at the temporal pole (Fig. 2), more significant relationship between subjective rating on the text flency and ISC at the right temporal pole (Fig. 5), and more significant ISFC associated with the temporal pole (Fig. 6) in fiction reading in our study corroborated these results.

The other area showing higher ISC in fiction reading was the fusiform gyrus, which plays a role in empathy. The left fusiform gyrus was also found with more significant ISFC during fiction reading (Fig. 6). Emotional empathy increases hemodynamic responses in the fusiform gyrus^41^. The left fusiform gyrus of speech listeners was also found to be activated more strongly when the speech speaker was visible from an ego-centric position, suggesting its involvement in empathy processing^42^. Anatomically, the size of fusiform gyrus has been found to correlate with total empathy score^43^. These studies functionally and structurally corroborate our results that across-subject correlated BOLD signals during fictional texts reading are localized to areas related to empathy processing.

In conclusion, we presented written narratives naturalistically to understand the neural substrates of reading comprehension. In particular, we used this paradigm to reveal brain areas modulated by text quality (human *vs*. machine translation) and content (fiction *vs*. news), two holistic features that would be difficult to study using more conventional paradigms. Contrasting between brain activity subserving reading different translations, we found right precuneus has significantly different brain response stability across subjects, potentially related to coherent narrative representations and contents over tens of seconds. While reading fictions elicits more stable brain responses at empathy related areas (temporal poles and fusiform gyrus). This experimental paradigm can be applied to study other complex aspects of texts to better understand the related neural processing in reading comprehension.

## Supplementary information

supplementary material
