# Supplementary material for: Differential brain mechanisms during reading human vs. machine translated fiction and news texts
Source: Sci Rep. 2019 Sep 13;9:13251. doi: 10.1038/s41598-019-49632-w (PMC6744568; doi:10.1038/s41598-019-49632-w)
Supplement: Supplementary file 1 — supplementary material [file 41598_2019_49632_MOESM1_ESM.pdf]

Differential brain mechanisms during reading human vs. machine translated fiction and news texts

Fa-Hsuan Lin[1, 2, 3], Yun-Fei Liu[1], Hsin-Ju Lee[1,2,4], Claire H. C. Chang[4], Iiro P. Jaaskelainen[3], Jyh-Neng Yeh[1], Wen-Jui Kuo[4]

1 Institute of Biomedical Engineering, National Taiwan University, Taipei, Taiwan
2 Department of Medical Biophysics, University of Toronto, Toronto, Canada
3 Department of Neuroscience and Biomedical Engineering, Aalto University, Espoo, Finland
4 Institute of Neuroscience, National Yang-Ming University, Taipei, Taiwan

Original articles

Fiction #1

Where Frogs Quack

It was one of the weirdest bits of news I've ever had to break. "Something unusual is infesting the earth under our apartment block," I told my neighbor. "I think it's a herd of cows." Below our building and the field nearby could be heard the unmistakable moo of large cattle. It went on for days. Yet none of my encyclopedias, nor that trusty compiler of oddities, the internet, had any information on "burrowing cows". The closest I could get was "ground beef".

The mystery was eventually solved by Georgina Noyce, a Hong Kong-based columnist who writes about animals. She told me that the Asiatic Painted Frog does not go "ribbit" like many frogs, but makes a "moo" noise like a cow. Echoing underground drains amplify their voices to make them sound loud and terrifying.

I once had a speaking engagement at a school in mainland China, and began an extremely long story with plot points that hinged on animal noises. In the tale, a chicken makes its normal "buk-buk-buk" noise, which is heard by the characters as "book, book, book." A frog makes its usual "ribbit-ribbit-ribbit" noise, which is heard as "read it, read it, read it."

I was well into the tale when I realized that the 900 kids listening to me had no idea what I was talking about. With horror I recalled that the sound chickens are perceived to make in China is not "buk-buk-buk" but "gordok, gordok, gordok." Worse, in that area, frogs quack like ducks. I once sat next to a busy frog pond in Guangzhou which sounded like a convention of Donald Duck impersonators.

Animal sounds are minefields for a travelling storyteller. In the Philippines, roosters go "tiktilaooo", in Mexico, "kikiriki", and in Portugal, "coco-ro-coco". The prize for "Most Accurate Sound" goes to Indonesia, where the sound is described as "kukuruyuuu". And "Least Accurate"? English speakers believe roosters wake up every morning and say "cock-a-doodle-do".

But Indonesians lose their crown for accuracy when it comes to frogs. They hear frogs go "tekotek, tekotek". Huh? English speakers redeem themselves when they describe pig-speech as "oink, oink". Compare that to the Japanese rendering of pig grunts as "boo-boo, boo-boo." I don't think so.

Dog-speak is a highly contentious issue. Indonesian hounds go "guk! guk! guk!" while Filipino ones go "aw! aw! au!" European ones go "wau! wau! wau!" but American ones go "woof-woof!" I think I would give the prize for accuracy to the Chinese, who claim dogs sound like, "houh! houh! houh!" and the least accurate to the British, who believe, incredibly that hounds go "bow-wow".

On my travels, I came across two books on this subject. Everywhere the Cow Says Moo! By Ellen Slusky Weinstein was charming but inaccurate. Bengali cows go “hamba” while Dutch cows say “boeh” according to a fun-with-the words book called The Meaning of Tingo by Adam Jacot de Boinod.

Are there animals which say the same thing around the world? Yes. Almost everywhere I’ve travelled, the sound a cat makes is described as “meow” or something similar (“miau” in German, “Ming” in Tagalog). Oddly, the speakers of Nahuatl, a language in Mexico, hear meow as “tlatzomia”. I blame it on the tequila.

Anyway, back to the scene at the school in China. So there I was, in mid-sentence, realizing that the story I was telling would make absolutely no sense to my audience. I had no idea what to do.

So I simply continued, leaping around and doing animal impressions, extending the story as far as I could. The children roared with laughter. The teacher told me afterwards: “The first time you came, you told them a story. That was OK. But this time you just talked like a crazy man, made no sense. I think they like this better.”

Fiction #2

Turning The Page

John Ribble, a soft-spoken gentleman responsible for testing at Godwin High School in Richmond, Virginia, held out my son's answers to a reading test. Louie, he told me, had an acute learning problem.

I protested, "I'd know if he was struggling that much. I'd be able to tell."

He pointed to one answer on a timed test that required more speed as it went along. Toward the end of the test, Louie had indicated that May was a day of the week. Ribble looked at me and said, "No one ever misses that question."

He pulled out old standardized test scores for Louie that showed wild inconsistencies. Such results, he said, and Louie's school record suggested not only that my son was dyslexic, but also that he likely had attention deficit disorder. Ribble said Louie wasn't just failing, he was suffering.

Louie was then a tall, creative 16-year-old whose wild, curly hair made him easy to find in a crowd. I'd raised him by myself almost from the time of his birth.

When Louie was in first grade, it became obvious that he had difficulty reading. To avoid having him labeled, I read everything to him. Every book he came in contact with, whether it was for school or fun, I would read to him. To entertain him, I created funny voices and made comments about the plot.

Through elementary and middle school, Louie grew into a thoughtful, intelligent, articulate boy who earned mostly B's but who had trouble comprehending the little he could read. No one else knew, and Louie and I rarely talked about it. I simply wouldn't accept that there was a problem.

In ninth grade, however, Louie's workload became so large that I couldn't keep up, could no longer read everything to him - and he was becoming too big and independent to want me to.

To prove his point, Ribble polled Louie's teachers and found out he had recently stopped turning in most of his homework. He showed me the scores, tapping the grade sheets with all the zeroes: "Luie is giving up," he said.

Ribble sensed that I was part of the problem. He was a representative of a public school system trying to do the right thing, trying to bring a parent out of denial. At long last, I stopped talking and began listening.

When I told Louie about the diagnosis, he didn't look hurt or confused. Instead, his face relaxed, and he shouted, "You mean I'm not stupid!"He was relieved.

I started to cry. "Were you worried too?"he asked.

I cried harder. By denying the truth to myself and thus keeping it from him, I had left Louie with the only other plausible answer he could come up with: He was dumb.

In a daze, I sat through the first meeting to design an individualized education program (IEP) for him. But over the next few months, the school officials and I mapped out a plan to address his weaknesses and bolster his strengths. The school paid for him to become a member of Recording for the Blind & Dyslexic, an organization that supplies CDs and books on tape from its library. He can use the service for life.

The hardest thing to do was to take a long look at my own behavior. While I had done a number of things right, I had fallen into the trap of trying to make Louie conform to my own definition of learning instead of being open to the idea that he would have his own path.

From that point, Louie began taking responsibility for himself. He is naturally talented in the visual arts. Though no one knows why, many kids with dyslexia have this gift.

Not long after he started his IEP, Louie got the first report card of his sophomore year. I tried to look both congratulatory and empathetic as I waited for him to tell me how he had done. I didn't want him to think I didn't expect much. Nor did I want him to conclude that I wouldn't be happy with whatever he had achieved. A tricky balance.

Louie had made the honor roll. A wave of relief came over me. He had stapled his report card down the middle to keep it open, and it already looked a little ragged. "You've been carrying it around?" I asked. "Yeah," he said, trying to sound casual.

"How about if I frame it?" I said, trying to do the same.

"Yeah," Louie said. "That's be good."

Good got better. Louie learned to love words, mixing and matching and stringing them together for others to read. He graduated from high school and is now at a community college in Chicago, studying to be a sports journalist - and still following his own path.

Fiction #3

<u>To Forgive Is Good</u>

Some years ago, a barrage of thumping, galloping noises routinely issued from the apartment upstairs as if baby elephants were competing in 50-yard dashes. I went up one day to politely inquire.

“No, nobody’s making any noise here,” the husband and wife both insisted. “It must be coming from elsewhere in the building.”

Two children about five years old, each holding soccer balls, stood right beside their parents.

“Could the thumping be your kids running around, perhaps playing soccer?” I asked.

“Oh, no, we never let the kids play in the house.”

For months, the pattern continued: the thumping and galloping above, our delicate check-in, the denial. It got so that every time I saw the couple, I glared without a word of greeting. When they moved out of the building, the thumping stopped.

I suppose I could have forgiven my neighbors this infraction and spared them the glare. After all, forgiveness is in, a trend spawning bestselling books, foundations, and research institutes. The notion has gone well beyond spiritual leaders advising that forgiveness is good for the soul and that hard feelings will turn us bitter and hostile. Now the medical community cites studies showing that forgiveness can prevent heart attacks, lower blood pressure, and even ease depression.

I may be outnumbered, but I still believe in the healing power of the grudge. I’ve deployed grudges with an equal-opportunity sense of fairness ---- against teachers and classmates, bosses and colleagues, family and friends. I’ve chosen to stop speaking to certain people permanently and occasionally even spoken ill of them ---- but more with incredulity than a sense of revenge. I’m neither proud nor ashamed. But I’ve discovered that nothing feels quite as satisfying as a grudge well nursed.

I had a boss who took a dislike to me from my first day on the job, even though she’d hired me. There were no complaints about my performance, but I later learned she’d lied to coworkers about me. Without explanation, she laid me off after only ten weeks, just before Thanksgiving. I had a family to support. Was I to forgive her? Should I now? Give me one good reason. My grudge against her balanced out that injustice, somehow righted the universe. It has kept me warm on many a cold night.

Is it just me? Under the new mandate of blanket absolution, should I forgive the cousin who invited us to dinner only to make an Amway pitch? Or the friend who sent me a public relations client and the harassed me for months for a 10 percent finder’s fee?

I’m not against forgiveness per se; I have forgiven people for rudeness as well as for deep misunderstandings and have done so without holding on to hard feelings. What

I deplore is the propaganda about forgiveness. No longer an option, forgiveness is an edict. Forgiving so democratically cheapens the very act.

A long-standing grudge suggests that we hold certain standards, that we respect ourselves enough to reject bad behavior. Failure to forgive can be just as righteous, just as honorable, as forgiveness itself.

When someone apologizes, however, with sincerity, not calculation, it can make a difference. I had a close friend in high school who ditched me after college and has avoided me for all the decades since. At our 15th high school reunion, I had the chance to ask him why. He said that I had always made him feel inferior, as if he gave offense. And he had a point: I'd made fun of him ---- I'd thought good-naturedly ---- until he withdrew. Face-to-face at our reunion, I apologized. He declined. I know how it feels to go unforgiven. And guess what? It feels deserved.

Fiction #4

<u>Threading the Needle</u>

Mama sat quietly in the corner of her bedroom, sewing a torn shirt. Her reading glasses flashed momentarily in the light when she looked up to see who had come in.

Across the room under an open window stood a large grey suitcase. Although it was framed by bright, mint-green walls and a curtain fluttered merrily over it, it was a lonely sight for me. For the suitcase and all the things inside it would be the only things from home that Mama would have with her when she left for Canada in a week's time.

Mama barely earned enough for our family as a Department of Health inspector in a small town in the southern Philippines. In addition to paying the regular bills and expenses, she insisted on sending her three children to a private Catholic school. But Mama was admirably hardworking. Each day she endured a 90-minute bus ride to her office and back again to our humble home in Davao City. Often, she travelled across rocky roads to faraway villages to do fieldwork, sometimes coming home sunburned and aching.

I was 11 years old. Mama decided to leave in 2001 for Toronto, where her brother lived, to take a job as a domestic helper. My brother Johannes, sister Jollibee and I would stay with our uncle and aunt.

The three of us were counting down the days, wondering when we would see Mama again after she left.

"I just wish she didn't have to go," I said.

"She wants to go. She wants to get away from you," Johannes would tease me. "You're so unbelievably stinky when you get home from school that she wants nothing more than to move to another country."

I would stick my tongue out at him, but I knew he was kidding. Mama wanted to improve our lives by working abroad. She wanted to be a good provider, a role she has shouldered since her marriage to our father crumbled when I was seven. After that, we never saw him or received any support from him.

Raising three kids on her own was extremely difficult. Sometimes during examinations, my siblings and I had to stand before the severe-looking nuns and beg them to allow us to take the exams even though our school fees weren't paid up.

I often wondered why Mama didn't send us to a public school instead. It would have spared her something like 3000 pesos ($64) every month. But I know exactly what she would have said if I had ever had the nerve to ask her: "Education is important. We may not be rich but at least I have given you that priceless gift."

Mama pulled me back from my thoughts as I stared at the suitcase in the corner. Holding out her needle and thread, she said, "Inday, could you please do it for me?"

I understood what she meant. She always asked me to put the thread through the

needle hole when she was sewing. Sometimes it annoyed me.
“Why always me?” I once demanded, after she had interrupted me while I was playing jackstones with my cousins.
“Because you have clearer eyesight,” Mama said.
“Well, Ate has clear eyesight,” I said, referring to Jollibee. “Why won’t you get her to do it?”
“Because you’re the youngest,” she simply replied.
Today, as I took the needle and thread from Mama, I noticed that her nose glistened with sweat from the effort of trying to do it herself. It took some time for me to thread the needle, but I knew that if I’d let Mama do it, she would have missed her flight to Canada before she actually succeeded.
I realized at that moment that threading the needle for Mama was a very small favor compared to what she had already done and will be doing for us. When I finished, I handed the needle back to her. I dared not look her in the eyes, because I knew what she would see in mine ---- sadness.
That feeling only grew worse as my gaze fell on the suitcase again, and then the needle in her hand. Without thinking, I said: “Who’s gonna put the thread through the needle for you in Canada?”
I wanted her to know that I would put every thread through every needle in Davao City just to make her stay, even if it did annoy me.
Tears started rolling down Mama’s cheeks. She reached out and pulled me into a tight embrace. I started crying too because at that moment, I knew what Mama’s answer was ---- no-one.
I realized that everything that she needed from home, she could carry not in a suitcase, but inside of her. I understood that all those tiring days Mama spent climbing hills through remote villages were nothing compared to what she could still do. She would be willing to go through a thousand needle holes if it meant a better life for us. I knew at that moment that even though Mama would be far away from us for years to come, our hearts would forever be sewn together.

News #1

China Strengthens Its Antitrust Push

When almost 100 government antitrust investigators simultaneously marched into four of Microsoft's offices across China last month, they were not looking for tea and gossip.

In what Microsoft characterized internally as "surprise visits," the agents from China's State Administration for Industry and Commerce interrogated a company vice president and other senior managers, copied contracts and downloaded large amounts of data, including emails and other internal communications.

The swoop on Microsoft stood out for its scale, but it was just one of dozens of similar actions across China recently that have set off alarms in boardrooms across the globe. Chinese regulators appear to be expanding enforcement of the antimonopoly law, and foreign companies fear that they could become easy targets for officials from agencies and local governments aiming to impress President Xi Jinping, the Communist Party leader who has promoted visions of patriotic resurgence and technological pre-eminence.

Foreign companies worry that the investigations could represent the rise of protectionism cloaked in regulatory impartiality but intended primarily to promote Chinese companies, especially the powerful state-owned companies. The government says that it is using the antimonopoly law, first established in 2008, to protect consumers.

"If China is going to be the third leg in the global antitrust regime, along with the U.S. and the E.U., and that's clearly coming, then the key question is, What sort of approach is China going to take?" John Frisbie, president of the US-China Business Council in Washington, said. "Is it going to be more the socialist state-run model, or is it going to be more of a market- and consumer-oriented model, or something in between? I don't think we know the answer yet."

Not everything in China is stacked against multinationals, some of which have their own political allies and business partners. Nor is China the only country where Microsoft and other companies have faced consumer ire and regulatory scrutiny. But multinationals appear to be facing new and substantial challenges across China.

"China has a very large bureaucracy, but each agency has its incentives and missions, so when they enforce the law, they try to maximize their own interests," said Angela Zhang, a lecturer in law at King's College London . "But I wouldn't underestimate the power of some really large multinational companies, because these companies are also very deep-pocketed and have very good connections in China." For now, though, Microsoft; the San Diego-based chip maker Qualcomm; Daimler, the German auto giant; and other formidable companies are on the

defensive.
Over recent weeks, investigators have been particularly busy — and public — in their efforts, setting a pattern of making abrupt searches of foreign companies' offices. These are reported by local media, confirmed by the companies and followed by the government's declaring that the companies are suspected of violating pricing, distribution and bundling rules.
On August 6, the State Administration for Industry and Commerce conducted a follow-up raid on two Microsoft offices in China and searched Accenture's offices in the city of Dalian. In early August, officials at the National Development and Reform Commission searched the Shanghai offices of Daimler, which makes Mercedes-Benz cars, and said they planned to fine Chrysler and Audi. On August 11, Audi said that in one province, the dealership network of its Chinese joint venture had broken national antitrust rules. A day later, General Motors said that it had been contacted by the Chinese authorities.
The reform commission enforces pricing violations of China's antimonopoly law and has been investigating how automakers price the spare parts that are sold by their distributors in China. In response, BMW said it would lower parts prices. Similar component price cuts have been made recently by Mercedes and Audi.
On August 13, Chinese news media said an adviser to a government committee had been dismissed, accused of accepting payments from Qualcomm, which is under investigation on suspicion of antitrust violations.
The company had made "large payments" to Zhang Xinzhu, an economist, while he was an adviser on an antimonopoly committee, reports said.
Among foreign companies, few would appear to be better connected in China than Microsoft. Its co-founder, Bill Gates, has met with Mr. Xi several times and he hosted Mr. Xi's predecessor, President Hu Jintao, at a 2006 dinner at his home in Medina, Washington.
It is not clear to Microsoft executives precisely how the Chinese authorities believe that they violated the country's law. Chinese officials have said publicly that the investigation is related to software compatibility, bundling and file verification issues for Windows and Office.
Early this month, The People's Daily, the chief newspaper of the party, told foreign companies to get used to tougher scrutiny: " Every kind of business should adjust its behavior and thinking to this new regulatory normal."

News #2

Creatures That Hide in the Open

The oceans, which make up more than 90 percent of the earth's livable space, are full of almost invisible animals.

To illustrate why, Dr. Sonke Johnsen, a professor of biology at Duke University in North Carolina, began a recent talk with a macabre scenario. Suppose just then a gunman burst into the room, shooting at the audience. Naturally, people would scramble for cover behind chairs and walls.

His point: There would be places to try to hide.

On land, many animals camouflage themselves amid the foliage and terrain; in coastal waters, sea creatures blend into the sand or find refuge among coral or rocks. But in the deep ocean, creatures floating in the water have nowhere to seek refuge.

Transparency is the most obvious strategy and the one Dr. Johnsen first began researching almost 20 years ago.

Transparency is not just a lack of pigmentation. Albinos, Dr. Johnsen points out, are not invisible; rather, the entire body must absorb or scatter as little light as possible.

Scattering is a challenge. When light passes into a material of a different index of refraction, which is often proportional to the density, part of the light reflects and part of it bends. That largely explains why one could search long and wide for a transparent cow or pigeon and not find one: The density of air is so much less than that of flesh that even a see-through terrestrial animal would probably be easily spotted from its reflections.

Water is much denser, and body tissues are roughly the density of water, greatly reducing the amount of scattering. But some organs are denser than others, and the transparent animals pack their insides differently to minimize the reflections.

Dr. Johnsen's measurements of the see-through creatures that he brought up from the depths found that 20 to 90 percent of the light passed through, undisturbed. "You could read a book through these animals," he said.

But transparency can complicate life, and transparent creatures near the surface could be sunburned, not only on the skin but inside, too. To protect themselves from ultraviolet light, "these guys basically have suntan lotion in their transparent tissues," Dr. Johnsen said.

Evolution has come up with two other forms of stealth technology: mirrors and biological light bulbs.

Some predators find their food by looking for silhouettes above. "You see many animals with upward-looking eyes, and even a squid with one big eye looking up and a 'normal' eye looking to the side," said Steven Haddock, a scientist at the Monterey Bay Aquarium Research Institute in Moss Landing, California.

The silvery sides of fish like herring and sardines are systems of mirrors: They reflect the down welling light, much the way a part of the sky is sometimes reflected by a glass skyscraper. Thus, a predator from below would see the blue water, not a fish, above.

Eric Denton, a British marine biologist, studied mirrored fish in the 1960s and figured out that the mirrors were vertical, maximizing the illusion.

The third strategy, called counter illumination, also seeks to mimic the down welling light. But instead of mirrors, the animal generates its own glow, much as fireflies do with light-producing organs known as photophores.

The animals employing counter illumination make sure the light they are producing is pointed downward.

“They don’t want light leaking out to the side and making them vulnerable, so they have lenses, mirrors and filters on their photophores,” Dr. Haddock said.

Some animals have evolved ways to defeat the camouflage. Species of squid and shrimp have eyes that can differentiate between the polarizations of light, something that many insects can do, but which people cannot do without polarized sunglasses.

Photons – particles of light – can be thought of as arrows with tail fins representing the oscillating magnetic and electric fields, and the polarization represents the orientation of the fields. To human eyes, the color of reflected light is unchanged. When reflected, the angle of the polarization changes.

As the sun moves across the sky, the polarization of light filtering down to the depths changes, and to an eye that can tell the difference between the polarizations, a mirrored fish suddenly sticks out.

“It turns out while the camouflage is really good, you can really break it with polarization vision,” Dr. Johnsen said.

That might, for instance, allow a squid to spot an approaching hungry tuna and flee.

“We’re just surrounded by an entirely mysterious world,” Dr. Johnsen said. “And the fact we can’t see it means we ignore it most of the time.”

News #3

A Way to Put Carbon Back Under the Ground

A new coal plant has been built here in the vast prairie to replace one that used to emit so much soot that clothes drying outside in the area would be covered with grit. But as with even the most modern coal plants, its smokestacks still emit enormous amounts of carbon dioxide, the invisible gas that is the main contributor to global warming. So this fall, a new maze of pipes and tanks will suck up 90 percent of the carbon dioxide from one of the boilers so it can be shipped out for burial, deep underground.

The effort will be the first major one of its kind at a power plant, the equivalent of taking about 250,000 cars off the road. And at least in theory, that carbon dioxide will be kept out of the atmosphere forever.

Worldwide, coal consumption in 2020 will be about twice what it was in 2000, according to the United States Energy Information Administration, and will continue to grow for decades.

"If you want to carry on using those fossil hydrocarbons, that means cleaning up their emissions," said Stuart Haszeldine of the University of Edinburgh. Capturing carbon, he said, "is the single best way of doing that."

Yet because it requires so much energy, sucking up carbon reduces a plant's ability to make electricity . There are basic questions of whether carbon dioxide can be safely stored underground. And the technology is expensive. Updating the Saskatchewan plant alone cost $1.2 billion .

In the pine woods of central Mississippi, another carbon-capture effort is taking shape, in a massive new power plant that will be fed a steady diet of coal from the strip mine next door.

Bruce Harrington, the operations manager, said an army of workers is cutting, welding and testing underground. The plant's owner, Southern Company, hopes to open the structure next year. But it is more complex than the Saskatchewan effort, and the price tag is now $5.5 billion.

The United States and other nations have helped some projects — Canada gave $220 million to the Saskatchewan plant's owner, SaskPower, and Southern Company received $270 million from the United States Department of Energy — but the costs are high enough that few other power companies have done much beyond study the concept.

The technology has been around for nearly a century, used at some refineries and other industrial plants, including large ones in Illinois, North Dakota, Canada and Norway.

But removing carbon dioxide from the swirl of gases unleashed at a power plant is

challenging. The equipment is enormous. At the Saskatchewan plant, called Boundary Dam, a liquid chemical latches onto carbon dioxide molecules after being sprayed onto a plume of combustion gases. The “stripper,” where the carbon dioxide is finally pulled away, is nearly 50 meters high .

In addition, efficiency is lost because some of the steam that would normally generate electricity goes to the stripper instead.

And a monstrous motor compresses the carbon dioxide — until it effectively becomes a liquid — for transport. All told, capturing the carbon dioxide at Boundary Dam will sap electricity generation by about 20 percent . Injecting liquids deep underground can present problems, too. Pumping wastewater from oil and gas production into the ground has been linked to small earthquakes in the United States.

The carbon dioxide could taint drinking water, or bubble up into the atmosphere, defeating the entire purpose.

Still, carbon dioxide has been buried around the world with few problems. In Norway, about a million metric tons have been stored every year since 1996, injected into sandstone about 900 meters beneath the North Sea. Most of Boundary Dam’s carbon dioxide will become a tool to extract and consume oil.

After being sold and shipped through a 60-kilometer pipeline to an oil field, the carbon dioxide will be pumped into old wells, where it will mix with the oil inside, making it flow better. The process is known as enhanced oil recovery . The oil and gas industry has done this for decades, mostly with naturally occurring carbon dioxide that accumulates underground. But each year in North America, more than 13 million metric tons of carbon dioxide from industry are used as well.

The practice could be expanded at many oil fields , experts say, potentially storing billions of metric tons of carbon dioxide and serving as a bridge to the day when it becomes necessary, and economical, to store the gas elsewhere.

The prognosis for carbon capture around the world is unclear.

If the United States moves forward with President Obama’s plans to cut carbon emissions, China and other countries may make bigger strides as well.

“How this will play out over time is hard to tell,” said Edward S. Ruben of Carnegie Mellon University in Pennsylvania. “Inevitably, there will be a balance between technological capability, cost and political realities.”

News #4

<u>Railroads Shift to Oil, Holding Up Grains</u>

The furious pace of energy exploration in North Dakota is creating a crisis for farmers whose grain shipments have been held up by a vast new movement of oil by rail, leading to millions of dollars in agricultural losses and slower production for breakfast cereal giants like General Mills.

The backlog is only going to get worse, farmers said, as they prepared recently for what is expected to be a record crop of wheat and soybeans.

“If we can’t get this stuff out soon, a lot of it is simply going to go on the ground and rot,” said Bill Hejl, who grows soybeans and wheat in a town near here.

Although the energy boom in North Dakota has led to a 2.8 percent unemployment rate, the lowest in the United States, the downside has been harder times for farmers who have long been mainstays of the state’s economy.

Agriculture was North Dakota’s Number 1 industry for decades, representing a quarter of its economic base, but recent statistics show oil and gas have become the biggest contributors to the state’s gross domestic product.

Railroads have long been the backbone of North Dakota’s transportation system and the most dependable way for farmers to move crops — to ports in Portland, Oregon; Seattle; and Vancouver, from which the bulk of the grain is shipped across the Pacific to Asia; and to East Coast ports like Albany, New York, from which it is shipped to Europe.

But reports the railroads filed with the government show that for the week that ended August 22, the Burlington Northern Santa Fe Railway — North Dakota’s largest railroad — had a backlog of 1,336 rail cars waiting to ship grain and other products. Another rail road, Canadian Pacific, had a backlog of nearly 1,000 cars.

For farmers, the delays often mean canceled orders from food giants that cannot wait weeks or months for the grain they need to make cereal, bread and an array of other products.

United States Agriculture Department officials recently said they were particularly concerned that Canadian Pacific would not be able to fulfill nearly 30,000 requests from farmers and others for rail cars before October.

“This rail backlog is a national problem,” Senator Heidi Heitkamp of North Dakota said. “The inability of farmers to get these grains to market is not only a problem for agriculture, but for companies that produce cereals, breads and other goods.”

A recent study conducted by North Dakota State University at Ms. Heitkamp’s request found rail congestion could cost farmers in the state more than $160 million because a local over supply of grain has lowered prices.

The study also found that farmers would lose $67 million in revenue from wheat, corn and soybeans from January to mid-April. Around $95 million more in losses are expected if farmers are unable to move their remaining crops.

Food companies say they are feeling the effects of the delayed shipments. General Mills, the Minnesota-based maker of Cheerios, the popular brand of oak cereal, told investors in March that it had lost 62 days of production — as much as 4 percent of its out-put — in the quarter that ended in February because of winter logistics problems, including rail-car congestion.

In its earnings report in August, Cargill, another Minnesota-based food giant, reported a drop in net earnings that it attributed in part to "higher costs related to rail-car short-ages."

Farmers and agriculture groups say rail operators are clearly favoring the more lucrative transport of oil.

Rail shipments of crude oil in North Dakota have surged since 2008, and the state now produces about a million barrels a day. About 60 percent of that oil travels by train from the Bakken oil fields in the western part of the state to far-away oil refiners. There are few pipe lines to ship it.

B.N.S.F. and Canadian Pacific maintain that their oil shipments have not replaced shipments of crops.

"Of course, the big difference in what we are shipping these days is oil," said Matthew K. Rose of B.N.S.F. "But we aren't favoring one type of product over another."

Nonetheless, B.N.S.F. is in-vesting about $400 million in North Dakota, to build additional tracks, hire new staff members and add rail cars. Mr. Rose said, "We're making this investment in our infrastructure to make sure that we get things back to normal."

Human translations

FH1

#01 青蛙都是「呱呱呱」？

現在想起來，下面是我跟別人說過最詭異的事：「我覺得在我們的公寓底下有些不尋常的東西，」我對鄰居說：「我覺得是一群牛。」在我們樓底下和周邊的田裡，可以清楚聽見只有大牲口才會發出的哞哞聲。這種情況持續了好幾天。不管是在我那些林林總總的百科全書裡，還是網際網路這個最可信賴的奇聞異事搜尋工具那兒，我都沒能找到關於「地下穴居牛」的資訊，我所能找到最接近的是「碎牛肉」。

謎團最後由香港的動物專欄作家喬吉娜·諾伊斯解開。她告訴我，亞洲錦蛙的叫聲不是常聽到的「麗比」，而是像牛一樣的「哞——」。聲音經過下水道的迴盪、放大，聽起來又響又駭人。

有一次我到中國大陸一所學校演講，說了個極長的故事，開場情節的「梗」就是動物的叫聲。故事中提到「布克，布克，布克」的雞叫聲，被其他動物聽成了「簿本，簿本，簿本」；青蛙一如往常叫著「麗比，麗比，麗比」，別的動物卻聽作「筆記，筆記，筆記」。

正當我全然入戲，卻發現 900 個聽講的孩子對我在說什麼毫無頭緒。我想起來中國人所理解的雞叫聲不是「布克，布克，布克」，而是「咯，咯，咯」，心頭頓時涼了半截。更糟的是，當地青蛙的叫聲像鴨子。我在廣州曾坐到滿佈青蛙的池塘邊，那股聲響宛如舉行唐老鴨模仿大賽。

描繪動物的叫聲，讓我們這種走訪各地的說故事表演者很是頭疼。菲律賓人認為公雞的叫聲是「提克提拉嗚」，墨西哥人說是「唧唧哩唧」，葡萄牙則說「叩叩嚕叩叩」。可能模擬得最像的是印尼人，他們把雞的叫聲形容為「咕咕嚕唷」。哪裡的人學得最不像呢？答案是英語系國家。他們形容公雞司晨的啼聲是「卡客——啊——嘟得——嘟」。

不過，換成青蛙，印尼人就不是口技冠軍了。他們形容青蛙的叫聲是「貼

叩貼，貼叩貼」。不會吧？在形容豬叫上，英國人總算扳回一城；英語裡豬是「歐因克，歐因克」叫的。日語模擬豬的咕噥聲為「噗——噗，噗——噗」，這個我可不敢苟同。

狗到底怎麼叫？不同的語言各執一詞。印尼語稱獵狗吠叫為「咕克！咕克！咕克！」，菲律賓人卻說「喔！喔！喔！」；歐洲人形容成「沃！沃！沃！」，可美國人說是「嗚夫——嗚夫！」我認為中國人的狗叫聲最逼真，他們說狗「汪！汪！汪！」地吠。至於最不像的是英國人，他們竟然認為獵犬的叫聲是「包——喔」。

在旅行途中我看到兩本以此為題的書。艾倫·斯拉斯基·萬斯坦寫的《天下牛兒一般「哞」！》，很有趣，但不盡正確。亞當·賈考特·德波伊寫過一本解釋文字趣味的書《「廷戈」是什麼意思》，書中提到孟加拉的牛叫聲是「漢吧」，而荷蘭的牛叫聲是「波耶」。

到底有沒有哪種動物的叫聲，舉世的說法都一致呢？有的。幾乎所有我到過的地方，貓的叫聲都被形容為「喵」，或是接近的聲韻（德語作「米奧」，菲律賓的塔加羅格語作「明」）。墨西哥講納瓦特爾語的人最古怪，竟能把「喵」聽成「特拉啾米亞」。我覺得他們是龍舌蘭酒喝多了。

言歸正傳，回到我在中國大陸的演講現場吧。口中的話說到一半，我察覺自己所講的內容對眼前的聽眾毫無意義，一時間，有點不知所措。

於是我索性把故事繼續說下去，一邊跳來跳去模仿各種動物的姿態，心想能講到哪兒算到哪兒。結果孩子們笑翻了天。後來，老師對我說：「你第一次來的時候給他們說了個故事，那次挺好的。不過這次你就像個瘋子，說了一大堆大家都不明白的東西。我覺得他們比較喜歡你今天的樣子。」

FH2

#04 翻開新的一頁

約翰・里勃說話溫文，是維吉尼亞州利奇蒙市歌文高中學生測試負責人，他拿著我兒子路易的閱讀測試答案卷給我看，說路易有嚴重的學習障礙。

我不以為然，回答說：「如果問題這麼嚴重，我不會不知道。我會看得出來。」

里勃指著計時測驗裡的一個答案：這種測試越到後面，學生得越快作答。路易在卷末把「五月」當作一星期的某一天。里勃望著我說：「從來沒有人答錯這一 題。」里勃拿出路易以前的統一標準測試成績，顯得非常參差。他說，從這些測試成績和路易的學校成績來看，我兒子不僅有閱讀困難症，還可能有注意力缺陷障礙。里勃說，路易不只是成績跟不上，而且深為學習所苦。

路易當時已經十六歲，個子高大，處事很有創意，長了一頭狂亂的鬈髮，在人群中顯得很突出。他幾乎從出生開始就由我獨力撫養。

路易小學一年級的時候，我已發覺他有閱讀困難。我怕他被視為問題學生，什麼都讀給他聽。凡是他要看的書，不管是課本還是休閒讀物，我全都讀出來給他聽。為了逗他開心，我讀時會用各種滑稽的聲音，並評論故事情節。

路易從小學升上中學，這時他已是個有心思、有智慧、表達力強的孩子，大部分科目都拿到 B 的成績，但他能夠自己閱讀的文字不多，理解也有困難。這件事我和路易很少談及，別人也不知道。我就是不肯承認自己的兒子有問題。

但是，路易到了九年級，課業多得我無法應付。我再也不能把什麼都讀給他聽。而他逐漸長大，越來越獨立，也不想繼續依賴我。

里勃為了證明他的觀點，還徵詢路易各科的老師，發覺路易近來大部分的家庭作業都沒有繳。他把路易的成績拿給我看，輕敲著滿是零分的成績單說：「路易漸漸心灰意冷了。」

里勃感到我也有問題。他代表公立學校，得把事情做好，必須令家長正視

問題所在。最後，我不再爭辯，開始聆聽。

我把里勃的判斷告訴路易，他沒有自尊心受損或不知所措的神情，反而面容一寬，高聲叫道：「你是說，我不是笨蛋！」他放心了。

我哭起來。

他問：「你和我一樣擔心嗎？」

我哭得更厲害。

我一直否認事實，路易於是也不知實情，能想到的唯一合理解釋就是：自己是個笨蛋。

我第一次出席為路易個人設計課程的會議，心中茫然，了無主意。但隨後幾個月，我和校方人員合力制定了一套計畫，補救路易的弱點，發揮他的長處。路易加入了「盲人及閱讀困難症患者專用錄音服務」，由學校付款。這個組織的圖書館有大量雷射光碟錄音和有聲書籍，供成員使用。路易可以終身使用其服務。

最困難的一件事，是好好檢討我自己的態度。我為路易做的事，不少都是對的，但未能避免一個錯誤，就是把自己對求學的看法強加在路易身上，不明白他應有自己的學習道路。

從此，路易負起了自己的責任。他對視覺藝術很有天分：患閱讀困難症的孩子不少都有這方面的天分，原因是什麼，沒有人知道。

路易接受特殊教育後不久，收到高二的第一份成績單。我等待他把成績告訴我時，努力擺出一副既高興又同情的神氣。我不希望路易覺得我對他期望不大，又不希望他覺得我不滿意他的成績，兩者之間很難掌握得恰到好處。

路易上了榮譽學生榜，我鬆了口氣。他在成績單中央釘了釘書針，不讓左右兩頁合起來。成績單看起來已經有點殘破。我問路易：「你一直把成績單帶在身邊？」

「是啊！」他盡量顯得若無其事。

我也用若無其事的語氣說：「我把這成績單裝框好不好？」

路易說：「好吧，好得很。」

路易的成績越來越好。他愛上文字，把文字重組、搭配、串聯起來給別人看。現在，他已經高中畢業，在芝加哥一所社區大學就讀，準備將來從事體育新聞的工作。那是他自己選擇的道路。

FH3

#06 寬恕是美德

幾年前，我住的公寓樓上經常傳來成串砰然作響的跑步聲，彷彿一群小象在進行五十碼短跑。

有一天我禮貌地上樓詢問，樓上那對夫婦斬釘截鐵地回答：「不是我們。我們沒發出任何聲音，聲音一定是從公寓裡別的地方傳出來的。」可是他們身旁明明就站著兩個年約五歲的孩子，手裡還各拿著一顆足球。

「有沒有可能是您的孩子在家裡跑來跑去的聲響，也許他們在踢足球？」我問。

「哦，不可能，我們從來不讓孩子在家裡踢球。」

這種對話模式持續了好幾個月：樓上咚咚奔跑，我們小心翼翼地探詢，鄰居堅決否認。後來終於演變為每次遇到那對夫婦，我總是一語不發地瞪著他們。等到他們一搬走，噪音也就消失了。

我當然也可以原諒鄰居這種不守規矩的行徑，而非怒目相視。畢竟，這年頭寬恕當道，許多暢銷書、基金會、研究機構都在大力宣揚寬恕精神。

宗教領袖倡言寬恕有助於靈魂的提升，而心懷不滿會使得我們充滿怨恨和敵意，但今天的寬恕已然超越性靈的範疇。醫學界引述研究報告，告訴我們寬恕可以預防心臟病、降低血壓，甚至減輕憂鬱症。

我可能勢單力薄，但我還是相信忿懣具有療癒的力量。不論面對老師或同學、老闆或同事、家人或朋友，我總是給怨恨一個公平、對等的機會。對於某些人，我選擇永遠不再和他們說話，甚至有時說他們的壞話，但與其說我這麼做是為了報復，不如說是出於不信任。我對此既不感到自豪，也不引以為恥。但我發現，沒什麼比心懷怨恨更令人心滿意足的了。

我之前有個老闆，她雖然雇用了我，但從一開始就不喜歡我。我的工作表現並無可議之處，但我後來才知道她會在我背後向同事搬弄是非。而我才做了十個星期，她竟然在感恩節前夕炒我魷魚，而且連個理由都沒有。我可是有家

要養的人啊。當時的我要原諒她嗎？我現在應該原諒她嗎？給我一個充分的理由吧。我對她的忿懣平衡了我所受的不公平待遇，以某種方式匡正了天理，在許多寒冷的夜裡溫暖了我。

難道只有我這麼想嗎？在一片「全然寬恕」的聲浪中，我應該原諒我表弟嗎？他請我們吃飯，結果真正目的是要推銷傳銷產品。我也該原諒我那個朋友嗎？他介紹了一個公關客戶給我，然後為了一成的介紹費騷擾我好幾個月。

我反對的並不是寬恕本身。我可以原諒別人粗魯的態度，原諒別人對我極度的誤解，而且事後不會耿耿於懷。我不贊成的是許多人提倡寬恕的做法。寬恕彷彿不再是種選擇，而是法令。一視同仁的寬恕貶低了這項美德。

持續不去的忿懣表示我們抱持某種標準，表示我們足夠尊重自己，能拒絕惡行惡狀的行為。無法寬恕可以和寬恕同樣正當，同樣值得尊敬。

然而，若有人的道歉是真心誠意而非出於算計，情況可能就會不一樣。我有位高中好友從大學畢業後就不再理我，而且幾十年來一直避免與我碰面。在第十五次的高中同學會上，我終於有機會問他原因。他說，我一直讓他感到自卑，好像他得罪了我似的。他說的話不無道理，我一直喜歡取笑他，還自以為不帶惡意，直到他終於與我疏遠。我在同學會上當面向他道歉，可是他拒絕接受。我現在知道不被寬恕是什麼滋味了。你猜怎麼著？我覺得我罪有應得。

FH4

#08 針線情

母親靜坐臥房一角，縫補破掉的襯衫。她抬起頭，看看是誰走進來了，眼鏡片在光下閃了一閃。

房間另一邊，在打開的窗下，有一個灰色的大手提箱；手提箱周圍是明亮的薄荷綠牆壁，箱子上方的窗簾隨風起舞，但這一切在我眼中卻有一種寂寥的感覺。再過一個星期，母親就要去加拿大，而她能從家裡帶去的，就只有這口箱子和裡面的東西。

我們住在菲律賓南部的一個小鎮，母親是衛生部督察員，薪水微薄，難以養家，除了支付日常的帳單和開銷，他還堅持送三個孩子上私立天主教學校。母親非常勤奮，每天辛苦搭九十分鐘的公車去上班，下班後再搭公車回到簡陋的家裡。我們的家就在達沃市。她還經常得走崎嶇的石頭路，到偏遠的小村莊檢查衛生情況，有時下班後，皮膚曬得通紅黝黑，渾身痠痛。

二OO一年，我十一歲，舅舅住在多倫多，母親決定去多倫多的家庭幫傭，我和哥哥約翰尼、姊姊茱莉比則會到姨丈、姨母家裏住。

我們三人都在倒數母親離去的日子，也不知道母親去後，什麼時候才會再見。

我說：「我真希望她不必去。」

約翰尼跟我開玩笑說：「她好想去，好想離開你。你放學回來時，臭得不得了，所以母親最想做的事，就是搬到國外去。」

我知道他在開玩笑，對他吐吐舌頭。母親想到國外工作，是要改善我們的生活，希望我們豐衣足食。我七歲那年，母親和父親分手，扛起養家的責任。從此我們沒有再見過父親，他也沒有負擔過任何費用。

母親一人養活三個孩子，極為辛苦。有時學校考試，我們兄妹的學費還沒繳清，只好站在一臉嚴肅的修女面前，請求她准許我們考試。

我常想，母親為什麼不送我們上公立學校？我們上公立學校，每個月就可

以省下三千披索（新台幣兩千元）。不過，即使我有膽子問，也知道她會怎樣回答：「教育很重要。我們雖然不富有，但至少我給了你們這件無價的禮物。」

我瞪著角落的皮箱，思潮起伏。母親把我從呆想中喚回，她一揚手中的針線說：「英戴，幫我一下好嗎？」

我知道她要我幫什麼忙。她做針線活兒的時候，總是要我把線穿入針孔，有時我真感到不耐煩。

有一次，我正在跟表姊妹玩拋石子遊戲，又被母親叫去幫忙，不禁抱怨說：「為什麼每次都叫我？」

母親說：「因為你的眼力好啊。」

我說：「阿茱的眼力也很好，為什麼不叫她？」

母親的回答很簡單：「因為你年紀最小。」

今天，我從母親手中接過針線時，看見她鼻頭微微發亮，那是努力穿針而淌的汗水。我也花了點兒時間才穿好，但我知道，要母親自己穿，穿好時，飛往加拿大的班機恐怕都要錯過了。

那一刻，我突然領悟到，和母親過去多年乃至未來多年為我們所做的一切相比，我為媽媽穿針，實在是微不足道的事。我穿好針，把針線還給母親時，不敢和她的視線接觸，她會看到我眼中的哀傷。

我望望那口手提箱，又望望母親手上的針線，心情更覺沉重，衝口而出說：「到了加拿大，誰幫你穿針啊？」

我希望母親知道，只要她留下來，達沃市上所有的針孔即使都要我穿，我也不會推辭，儘管我不喜歡穿針。

母親哭起來，伸出手，緊緊抱住我。我也哭了。我知道媽媽的回答是：沒有人可以幫她。

我還知道，母親真正要從家裡帶走的東西，無法裝在那口手提箱裏，只能藏在心中。我知道，母親跋涉偏遠山村的日子雖然苦，但她日後的工作會更苦。為求我們生活過得好一點兒，她甘心穿過一千個針孔。那一刻，我心裡明

白，母親和我們雖然會分隔多年，但是大家的心將永遠縫在一起。

NH1

#02 中國大陸加大反壟斷動作

將近 100 名官方反托辣斯調查員上個月突然同時進入微軟公司設在中國大陸的四個據點時，可不是去喝茶聊天的。

在微軟內部稱為「突然造訪」的行動中，這些來自大陸國家工商行政管理總局的調查員盤問微軟的一名副總裁與其他高級主管，影印合約並下載大量資料，包括電子郵件與其他內部通訊。

此次針對微軟的突襲檢查規模浩大，卻只是大陸最近所採取引起全球各國大企業恐慌的許多類似行動之一。大陸主管機關似乎正擴大落實反壟斷法，外國企業則擔心，它們隨時可能成為大陸各主管當局與地方政府官員的下一波目標。身兼中國共產黨領導人的大陸國家主席習近平提倡愛國主義與科技卓越，這些官員試圖讓他留下深刻印象。

外國企業擔心，這波調查可能代表一種保護主義的崛起，這種保護主義以公平管理為名，主要目的則在促進大陸企業，尤其是國營企業的利益。大陸當局則表示，要用 2008 年開始施行的反壟斷法保護消費者。

華府「美中貿易全國委員會」總裁傅里斯比說：「如果中國大陸是美國、歐盟之外，全球反壟斷體系的第三隻腳，而這顯然已逐漸成為事實，則關鍵問題是，中國大陸可能怎麼做？會不會是社會主義的國家主導模式？或是偏向市場與消費者導向的模式？又或介於兩者之間？我認為，我們還不知道答案。」

部分跨國企業有它們自己的政治盟友與商業夥伴。大陸並未事事針對它們，也不是微軟與其他大企業引起消費者不滿與主管機關監視的唯一國家。然而跨國企業似乎已在大陸各地面臨多項新挑戰。

倫敦國王學院法學講師張湖月說：「中國大陸擁有非常龐大的官僚體系，然而每一個政府機構各有不同的動機與任務。當它們執法時，自然會試圖將自己的利益最大化。然而我不會小看部分大型跨國企業的實力，因為這些企業同樣口袋很深，在中國大陸也有很好的人脈。」然而目前，微軟、總部位於美國

加州聖地牙哥市的晶片製造商高通公司、德國汽車巨擘戴姆勒公司與其他大企業暫時退居守勢。

最近幾周以來，調查員顯得尤其忙碌，試著公開確立突檢外國企業辦公室的模式。在地媒體報導這些事件，受到突檢的企業予以證實，大陸當局隨後則聲稱，這些企業疑似違反有關定價、配銷與綁售的法律。

8月6日，大陸國家工商行政管理總局對微軟在大陸的另二據點施以後續突檢，同時搜查埃森哲諮詢公司的大連辦公室。八月初，大陸國家發改委官員搜查賓士汽車製造商戴姆勒公司的上海辦公室，同時揚言對克萊斯勒與奧迪汽車開罰。奧迪汽車8月11日表示，它在大陸某一省的合資經銷網絡違反大陸的反托辣斯法律。一天後，通用汽車表示，大陸主管當局曾經與它接觸。

發改委負責處理與大陸反壟斷法有關的違規定價案例，而且一直在調查汽車製造商如何為它們透過大陸經銷商銷售的汽車零組件定價。BMW汽車表示，它準備降低零組件定價。賓士與奧迪汽車最近也公布類似的零組件降價方案。

8月13日，大陸媒體報導，官方某委員會的一名顧問因為涉嫌收受高通公司賄賂而被解職，該公司則因被指違反反托辣斯法而正在接受調查。

報導說，高通公司曾把「巨款」交給時任某一官方反壟斷委員會顧問的經濟學者張昕竹。

論到外國企業在大陸的的人脈，微軟公司罕有其匹。該公司共同創辦人比爾蓋茲曾經多次會晤習近平，而且曾於2006年在他位於華盛頓州麥迪納的私宅以晚宴款待習近平之前的大陸國家主席胡錦濤。

微軟高級主管無法確定的是，大陸當局如何認定他們違反大陸的法律。大陸官員曾經公開表示，調查與微軟視窗、Office軟體的相容性、綁售、檔案確認問題有關。

本月初，中國共產黨的主要報紙人民日報呼籲外國企業適應更嚴格的監督。它說：「各行各業都應該調整行為與思考模式，以配合新的管理常態。」

NH2

#03 海洋動物藏身於無掩蔽處

海洋占地球可居住空間的 90%以上，其中充滿各種幾乎不可見的生物。

為了證明箇中原理，美國北卡羅來納州杜克大學生物學教授江森最近以一種駭人的場景展開對話。假設一名持槍男子闖進這個房間，向著聽眾開槍；人們當然會躲在椅子與牆壁後尋求掩護。

他的論點：總會有那麼些地方可以躲藏。

在陸地上，許多動物在樹葉與地形之間偽裝自己。在沿海水域，海生動物融入沙子或在珊瑚、岩石之間尋求掩護。然而到了深海裡，浮游在水中的那些動物可就無處可以藏身了。 透明是最明顯的一個策略，也是江森博士將近 20 年前開始率先研究的重點。

透明不只是沒有天然色素那麼簡單。江森博士指出，白化並不是不可看見。要不被看見，整個身體必須盡可能把吸收或散射的光減到最少的程度。

如何散射是個挑戰。光進入折射指數（通常與密度成正比）不同的物質時，部分的光會反射，另一部分則彎曲。這足以解釋，為什麼一個人就算費盡功夫也找不到一隻透明的牛或鴿子。由於空氣的密度遠低於肌肉，即使真有一隻透明的陸地動物，只怕也會因為反射作用而輕易被人看見。

水的密度可就大多了，而身體組織的密度則與水相當，以致散射的光量大幅降低。然而部分器官的密度大於其他器官，透明的動物會以不同的方式包裝牠們的內臟，將反射降到最低限度。

江森博士測量他取自海洋深處的透明動物之後發現，有 20%到 90%的光線可以不受阻擋直接穿透。他說：「你可以隔著這些動物閱讀一本書。」

然而話說回來，透明也使得這些動物要活命變得更為困難。接近水面的透明動物可能曬傷，不只是皮膚，體內也會。江森博士說，為了讓自己不至於因為紫外線而受傷，「基本上來說，這些傢伙的透明組織裡含有天然防曬液」。

演化造就另外兩種隱形技術：鏡子與生物燈泡。

部分掠食者搜尋在自己上方的側影輪廓，據此找到食物。加州莫斯碼頭蒙特利灣水族研究所的科學家哈多克說：「許多動物具備往上看的眼睛。甚至有一種魷魚的一個大眼睛會往上看，另一個『正常』的眼睛則看著側邊。」

鯡魚、沙丁魚等魚類的銀色光澤側邊有如一排鏡子。它們能夠反射下降的光，就像一棟玻璃帷幕大樓有時候會反射一部分的天空。換言之，位於下方的掠食者只會看到湛藍的海水，卻看不到一條魚。

英國海洋生物學家丹頓 1960 年代研究有銀色光澤側邊的魚類後發現，這些鏡子呈垂直狀態，導致幻覺最大化。

第三種策略名為反照明，同樣意在模仿下降光。然而這些動物不使用鏡子，而是自體發光，就像螢火蟲以發光器官發光。

採用反照明方法的動物會確定牠們製造的光往下。

哈多克說：「牠們不希望因為光漏向側邊而使自己陷入危境。在這種情況下，牠們的發光器官還配備透鏡、鏡子與濾光器。」

有些動物演化出破解偽裝的方法。有些魷魚與蝦子的眼睛可以分辨光的偏振。許多昆蟲也有這種能耐，人類則必須借助於偏光太陽眼鏡才做得到。

我們可以把光子比擬為配置尾翼的箭。這些尾翼代表不斷振盪的磁場與電場，偏振則代表它們的定位。對人類的肉眼而言，反射光的顏色不會改變。一旦反射，偏振的角度就會改變。

太陽在天空移動時，過濾到海洋深處的光偏振會隨之改變。對能夠分辨偏振差別的眼睛而言，一條反射的魚就會變得明顯可見。

江森博士說：「偽裝固然很好，偏振的視覺卻可以破解。」

舉例來說，這或許可讓一隻魷魚察覺一隻飢餓的鮪魚正在接近，及時逃走。

江森說：「我們被一個完全神秘的世界包圍，看不到它意味我們多數時候忽略了它。」

NH3

#05 把碳封存在地下的方法

加拿大薩斯克徹溫省伊斯得芬的大草原上已建妥一座燃煤發電廠，取代曾經排放大量煤灰，讓戶外晾曬的衣物蒙上一層塵粒的舊發電廠。

燃煤電廠再現代化，煙囪還是會排放大量二氧化碳。這種看不見的氣體是促成全球暖化的關鍵。新的輸送管與儲存槽今秋將自其中一個鍋爐吸取九成二氧化碳，運出並深埋地底。

這將是發電廠首次做此類重大嘗試，相當於減少 25 萬輛汽車的排放量。至少在理論上這些二氧化碳永遠不會逸入大氣層。美國能源資訊局表示，2020 年全球煤消耗量預估會是 2000 年的兩倍，其後數十年還會繼續增加。

愛丁堡大學的哈塞爾丁說：「若要繼續使用這些化石碳氫化合物，這意味你必須清理它們的排放物。捕集碳是最好的方法。」

然而捕集碳耗費大量能源，會使發電廠的發電量減低。二氧化碳能否安全儲存於地底，也還有些基本問題尚待解答，而且技術所費不貲。僅是在薩斯克徹溫興建另一座發電廠就耗資 12 億美元。

在密西西比州中部的松林，另一項捕集碳的嘗試正在進行。一座新建的大型發電廠所需的煤將由毗鄰的露天礦場供應。

作業經理哈靈頓表示，工人正在地面下切割、焊接及測試。業主南方公司希望這座設施可於明年啟用。然而這項計畫比薩斯克徹溫的複雜，目前估計的成本是 55 億美元。

美國與另一些國家已經贊助一些類似的方案。加拿大政府補助經營薩斯克徹溫發電廠的薩斯克電力公司 2.2 億美元，美國能源部則補助南方公司 2.7 億美元。然而由於成本太高，絕大多數電力公司僅止於研究這個構想而已。

必要的技術問世已將近一個世紀，主要用於一些煉油廠與其他工廠，包括伊利諾州、北達科他州、加拿大與挪威的一些大型工廠。

然而從發電廠所排放的各種氣體中取出二氧化碳卻是個挑戰。必要的設備

體積龐大。在名為「邊界水壩」的薩斯克徹溫發電廠，一種液態化學劑噴向一縷燃燒氣體之後，就會與二氧化碳分子結合。最後取走二氧化碳的清除器高度將近 50 公尺。

此外，效率也會折損，因為通常用於發電的蒸汽有一部分也會逸入清除器。

一部巨大的馬達壓縮二氧化碳，直到它成為便於運送的液態。整體而言，邊界水壩捕集二氧化碳大約會使發電量減少 20%。把液體注入地底深處也會產生一些問題。將開採石油、天然氣所產生的廢水注入地底經證明與美國的一些輕微地震有關。

二氧化碳可能汙染飲用水或逸出並進入大氣層，導致整個計畫完全失敗。

實際上，二氧化碳已在全球各地掩埋，而且並未產生太多問題。在挪威，1996 年起，每年約有 100 萬公噸的二氧化碳注入北海海底下約 900 公尺處的沙岩層。邊界水壩的二氧化碳大多會成為開採並消耗石油的工具。

二氧化碳賣出並經由一條 60 公里長的管路輸送到一處油田之後，打入舊油井，與裡面的石油混合，提高石油的流動性。這項流程名為強化採油，在石油、天然氣業已行之數十年，大多是使用在地底自然累積的二氧化碳。不過在北美地區，每年平均也會使用超過 1300 萬公噸工業界產生的二氧化碳。

專家說，這種流程可在許多油田推廣，可能足以儲存數十億公噸的二氧化碳，同時充當有朝一日必要且具有經濟效益時，在其他地區儲存二氧化碳的銜接階段。

捕集二氧化碳的全球性前景仍不明朗。

如果美國總統歐巴馬的二氧化碳減排計畫得以落實，中國大陸與其他國家也可能大步跟進。

賓州卡內基美隆大學的魯本說：「這件事會如何發展很難說。技術、成本與政治現實之間必將取得平衡。」

NH4

#07 鐵路改運石油 不利農民

美國北達科他州近年來積極開採能源，對因為鐵路改運石油而導致穀物運輸受到延誤的農民構成危機，不但造成可觀的農業損失，早餐麥片製造大廠如通用磨坊等公司，生產也受到波及。

農民說，由於他們最近準備迎接創紀錄的小麥與大豆收成，存貨積壓只會更嚴重。

在北達科他州法戈市附近小鎮種植大豆與小麥的赫傑爾說：「如果不能盡快把收成送出去，它們會爛在地上。」

能源生產欣欣向榮使北達科他州失業率降至全美最低的 2.8%，壞處是，農民處境變得更艱困。

農業一直是該州經濟的支柱，多年來是它的頭等產業，占全州經濟基礎的四分之一。然而最近公布的數據顯示，石油與天然氣已在該州內部生產總值中占有最大比率。

長久以來，鐵路是北達科他州運輸系統的骨幹，也是農民運送收成的最可靠工具。他們利用鐵路將收成送到奧勒岡州波特蘭、西雅圖與加拿大溫哥華的港口，再以貨輪橫越太平洋轉送亞洲，或者運到紐約州奧爾巴尼等地的美國東岸港口，再以貨輪轉送歐洲。

然而根據鐵路公司向主管當局提交的報告，至 8 月 22 日為止的一周內，北達科他州最大鐵路貨運公司柏靈頓北方聖大非鐵路（BNSF），等待運送的穀物與其他產品多達 1336 節鐵路貨運車廂。另一家鐵路公司加拿大太平洋也有將近 1000 節車廂的貨物等待運送。

對農民而言，延誤往往意味食品大廠取消訂單，因為它們無法等待用於製造麥片、麵包及各種產品所須的穀物數周或數月之久。

美國農業部官員最近說，他們特別擔心加拿大鐵路公司無法在十月前，滿足農民與其他產業領域對將近三萬節貨運車廂的要求。

北達科他州選出的聯邦參議員海蒂．海特坎普說：「鐵路運輸延誤是全國性的問題。農民無法將收成送到市場不僅對農業構成問題，對生產麥片、麵包與其他產品的企業亦然。」

北達科他州州立大學最近受海特坎普委託進行的一項調查顯示，鐵路運輸延誤可能使該州農民至少損失 1.6 億美元，因為當地穀物過量抑低價格。

調查還發現，一至四月，農民將因為小麥、玉米與大豆而減收 6700 萬美元，而如果農民無法將剩餘的收成運送出去，可能另外虧損 9500 萬美元。

食品公司表示已經開始感受到穀物延誤運送的後果。總公司設在明尼蘇達州的通用磨坊公司生產廣受歡迎的「歡樂穀」牌早餐燕麥片，三月向投資人表示，受到包括鐵路運量不足等冬季運輸問題影響，在截至二月為止的一季，已經耽誤 62 天的生產，相當於總產量的 4%。

另一家同樣位於明尼蘇達州的食品大廠嘉吉公司則在八月發表的營利報告中指出，淨收益已經減少，原因之一是「貨運車廂短缺導致成本增加」。

農民與農業團體說，鐵路公司明顯偏好更有利可圖的石油運輸。北達科他州原油的鐵路輸送量 2008 年開始大增，該州目前每天大約生產 100 萬桶，其中約六成透過鐵路自該州西部的巴肯油田送到相隔遙遠的煉油廠。輸油管不多。

BNSF 與加拿大太平洋鐵路公司說，它們運送石油並未影響農作運量。

BNSF 的羅斯說：「當然，我們最近運送貨品上的重要差異是運送石油，不過我們並未因為某種產品而犧牲另一種產品。」

儘管如此，BNSF 已在北達科他州投資大約四億美元，準備鋪設更多的鐵軌，雇用新人力與增加貨運車廂的數目。羅斯說：「我們對基礎設施注資的主要目的是，確保一切回到正軌。」

Machine translations

FM1

#01 青蛙都是「呱呱呱」？

這是新聞，我曾經不得不分享最古怪的之一。「一些不尋常的為害我們的公寓樓在地下，」我告訴我的鄰居：「我認為這是一群奶牛。」下面我們的建設和現場附近區塊可以聽到大牛的哞哞無誤。這個狀況持續了幾天。然而，沒有我的任何百科全書，也不是那個值得信賴的奇怪的現象編輯者，互聯網，有對「穴居牛」的任何信息。最近我得到的是「碎牛肉」。

謎最終被喬吉娜・諾伊斯，香港為基礎的專欄作家誰寫關於動物的所解決。她告訴我說，亞洲圖畫青蛙不走「麗比」像許多青蛙一樣，但發出「哞哞」的噪音，就像一頭牛。呼應地下水渠放大自己的聲音，使他們的聲音響亮而恐怖。

我曾經有一個公開演講，在一所學校在中國大陸，並開始一個非常長的故事情節絞鏈連接在動物的聲音。在故事中，一隻雞製作其正常「布克，布克，布克」的噪音，這是被其他角色聽取成「簿本，簿本，簿本」一隻青蛙製作其一貫的「麗比，麗比，麗比」噪音，這是被聽到當作「筆記，筆記，筆記」

我順利進入故事時，我突然意識到，九百個孩子聽我不知道我在說什麼。跟著驚恐我記得的聲音雞被認為在中國沒有「布克，布克，布克」，但「咯，咯，咯」。更糟的是，在這區域，青蛙呱呱就好像鴨子一樣。有一次，我坐在旁邊一個繁忙的蛙池在廣州這聽起來像是唐老鴨模仿者的集會。

動物的叫聲是四處旅行故事講述者的雷區。在菲律賓，公雞走「提克提拉嗚」，在墨西哥，「唧唧哩唧」，而在葡萄牙，「叩叩嚕叩叩」。為「最精確的聲音」該獎項去印尼，那裡的這個聲音被描述為「咕咕嚕唷」。和「最不準確」？講英語的人認為公雞每天早上醒來，說：「卡客——啊——嘟得——嘟」。

不過，印尼人失去冠冕的準確性，當涉及到青蛙。他們聽到青蛙去「貼叩貼，貼叩貼」。咦，什麼？講英語的人們自我救贖，當他們形容豬講話為「歐因

克，歐因克」。與此相比，日本人把豬的呼嚕聲渲染為「噗——噗，噗——噗」，我不這麼認為。

狗說話是一個非常有爭議的問題。在印尼獵犬去「咕克！咕克！咕克！」，而菲律賓的人去「喔！喔！喔！」，歐洲的人去「沃！沃！沃！」，但美國的人去「嗚夫——嗚夫！」我想我會給予獎精確到中國，誰主張狗聽起來就像「汪！汪！汪！」和最不準確的英國，誰相信，非常不可思議，居然是獵狗去「包——喔」。

在我的旅行，我在兩本書來到這個問題。《無處不在的牛說哞！》由艾倫・斯拉斯基・萬斯坦是迷人的，但不準確。孟加拉母牛走「漢吧」，而荷蘭奶牛說「波耶」，根據一個有趣的和文字樂趣書本名為《廷戈的意義》由亞當・賈考特・德波伊。

是否有動物的說話，在世界各地是同樣的事情？是的。幾乎我旅行走過的每處，一隻貓發出的聲音都被描述為「喵喵」或類似的東西（「米奧」在德國，塔加羅格語是「明」）。奇怪的是，納瓦特爾語，在墨西哥語，說話的人聽到「喵喵」卻當作「特拉啾米亞」。我責備它於龍舌蘭酒。

總之，回到現場的中國學校。所以我是在那裡，在中間的句子，意識到這個故事我正在說的會使完全沒有意義對於我的觀眾。我不知道該怎麼辦。

所以我乾脆繼續，周圍四處跳躍，表演動物的印象，據我可以盡量遠延伸的故事。孩子們哄堂大笑。老師後來告訴我：「你第一次的時候來，告訴他們一個故事。這是不錯的。但是這個時候你剛才說到像一個瘋狂的人，沒有任何意義。我覺得他們喜歡這個更好的。」

FM2

#04 翻開新的一頁

約翰・里勃中，說話輕聲細語的紳士負責測試的歌文高中在維吉尼亞州利奇蒙市，伸出我兒子的考卷針對回答了閱讀測試。他告訴我，路易有一個尖銳的學習問題。

我不滿，回答他說：「我會知道如果他很吃力的了。我可以看出來。」

他指著一個答案的定時測試，需要更快的速度當它進行下去。對測試的結束時候，路易曾表示，五月是一周中的一天。里勃看著我，說：「從來沒有人錯過了這個問題。」

他掏出舊的標準化考試成績的路易是表明野生不一致。這樣的結果，他說，和路易的學歷表示不僅是我的兒子有閱讀障礙，也使他有可能有注意力缺失症建議。里勃說路易不只是正在不及格，他很痛苦。

路易是那麼高大，很有創意的十六歲，野又捲曲的頭髮使他很容易被找到在人群中。我幾乎從他出生時就獨自撫養了他。

當路易是在小學一年級，很明顯，他是有閱讀困難的。為了避免他被貼上標籤，我讀給他一切。每本書他進來接觸的，無論是在學校還是好玩，我就念給他聽。為了娛樂他，我創建了有趣的聲音，而且提出意見針對劇情。

經過小學和中學，路易成長為一個體貼，聰明，口齒伶俐的男孩，誰贏得大部分是 B 的成績，但誰遇到了麻煩理解他能讀懂的那一點點。沒有人知道，而路易，我很少談論它。我根本就不承認有問題。

在九年級，然而，路易的工作量變得如此之大，我無法跟上，再也無法閱讀給他的一切。而他變得過於龐大，更加獨立而不要我這麼做。

為了證明自己的觀點，里勃調查路易的每科老師，發現他最近停止了繳交，在大多數他的回家功課。他給我看那分數，拍打成績單與所有的零：「路易正在放棄，」他說。

里勃感覺到我是問題的一部分。他是一個公立學校系統試圖做正確的事的

代表，試圖把父母帶出否定行為。終於，我不再說話，開始聽。

當我告訴路易診斷一下，他沒有看起來受傷或混淆。相反，他的臉放鬆了，他喊道：「你的意思是我不傻！」他才鬆了口氣。

我開始哭了起來。「你是否也有擔心過？」他問。

我哭得更難。通過否認事實對自己，因而從他身上保持它，我已經離棄路易，唯一的其他合理的答案，他能想出：他是一個笨蛋。

在一個發呆中，我在第一次會議上坐著來設計個性化教育計劃給他。但在接下來的幾個月裡，學校的教職員和我制定了一個計劃，以解決他的弱點，並加強他有優勢的地方。學校為他付錢，使他成為記錄盲人和閱讀障礙，一個供給從它的磁帶庫的 CD 和書籍的組織的成員。他可以使用該服務為一生。

最難做的事情是需要很長的看看我自己的行為。雖然我做了一些正確的事情，我已經陷入了陷阱，試圖讓路易符合我自己的學習，而不是持開放態度的想法的定義，他將有他自己的道路。

從這一點來說，路易開始接受為自己的責任。他是在視覺藝術，生來就有才華。雖然沒有人知道為什麼，很多孩子有讀寫障礙的都有這份禮物。

他開始了他的特殊教育後不久，路易得到了他高二的第一份成績單。我試著看起來同時祝賀和同情，因為我等著他告訴我他是如何完成的。我不想讓他覺得我沒期望太多。我也沒要他得出結論，我也不會高興什麼，他取得的成就。這是一個微妙的平衡。

路易做了光榮榜。放鬆的波浪征服了我。他釘了成績單攔腰保持打開狀態，它已經顯得有點粗糙。「你一直背著它在嗎？」我問。

「是啊，」他說，試著聽起來休閒。

「怎麼樣，如果我裱框呢？」我說，試圖做同樣的。

「是啊，」路易說：「這是很好的。」

好的得到更好。路易學會了愛單字，混合和匹配，並串在一起給別人看。他高中畢業，現在在芝加哥一所社區學院，學習做個體育記者——現在依然按

照他自己的路。

FM3

#06 寬恕是美德

幾年前，一連串重擊的，奔騰從我住在的公寓樓上經常發出噪音，就好像嬰兒小象被在比賽五十碼短距離衝刺。我去了樓上一天禮貌地詢問。

「沒有，沒有人在這裡做任何噪音」，夫妻兩個都很堅持這個說法：「它必定是從其他地方的在建築之中來了。」

有兩個孩子，大概五歲左右，各自拿著足球，就站在他們身旁，他們的父母。

「請問碰撞聲可能是你們的孩子跑來跑去，也許在踢足球？」我問。

「哦，不會，我們從來沒有讓孩子們在屋子裡玩。」

幾個月來，這樣的模式繼續下去：重擊的聲音和疾馳在上面，我們的細膩向他們調查狀況，被他們否定。它得到了於是讓我每次看到這對夫妻的時候，我怒視著不說打招呼的話。當他們搬出了大樓，重擊的聲音停止。

我想我應該要可以原諒我的鄰居本次違規，並倖免他們的那個怒視。畢竟，寬恕是，這一趨勢，衍生出來很多暢銷書，基金會和研究機構。這個概念已經遠遠超越精神領袖建議說寬恕是對靈魂良好的，而堅硬的感覺會變成我們的痛苦和敵意。現在醫學社群引用的研究顯示，寬恕可以預防心臟病發作，降低血壓，甚至緩解抑鬱症。

我可能寡不敵眾，但我仍然相信，在鬧脾氣的有療癒力量。我已經部署了給積怨有公平的機會均等的意義——對老師，同學，老闆和同事，家人和朋友。我選擇停止永久向某些人發言，有時甚至說出他們難聽的——但更多與不相信而不是報復的感覺。我既不驕傲，也不慚愧。但是我發現沒有什麼感覺相當的滿意的像鬧脾氣也調養好的。

我有一個老闆誰拿了對我的不喜歡，從我第一天上班，雖然她已經僱傭了我。沒有人抱怨我的表現，但後來我才知道她騙了關於我的同事。沒有任何解釋，她只是後十個禮拜把我給遣散掉，在感恩節之前。我有一個家庭。難道我

原諒她？我應該現在原諒她呢？給我一個很好的理由。我對她的平衡來自不正義而鬥氣，不知如何地糾正了我的宇宙。它讓我溫暖了許多一個寒冷的夜晚。

難道僅僅是我嗎？在全方位的赦免的新的命令，要我原諒誰請我們吃飯，結果是在做安利推銷的表妹？或者誰送我一個公關客戶端，後來騷擾我很多月為了 10％中間人佣金的朋友嗎？

我不反對寬恕這個事情本身；我已經原諒人的無禮，以及深的誤解，沒有堅持著一種硬的感覺，已經這樣做了。我感到遺憾是關於寬恕的宣傳。變成不再是一種選擇，寬恕是一項法令。寬容全部所有人貶低了這個非常行為。

一個長期存在的不滿意感覺表明，我們擁有一定的標準，我們尊重自己，足以拒絕不良行為。如果不原諒，一樣可以理直氣壯，也是有光榮，就像寬恕一樣好。

當有人道歉，但是，是以真誠表現，沒有背後打算，它可以有所作為。我在高中的一位密友誰上大學之後拋棄了我，自從避免了我之後所有的幾十年。在我們的第 15 屆高中同學聚會，我有機會問他為什麼。他說，我一直讓他感到位置低下，好像他給了冒犯的行為。他說話有道理：我曾經取笑他——我還以為本意上是好的——直到他退出了。臉對臉，在我們的聚會，我向他道歉。他拒絕了。我知道那是什麼感覺去不被原諒。你猜怎麼著？感覺是應該得到的。

FM4

#08 針線情

媽媽在她的臥室的角落靜靜坐著，縫合撕裂的襯衫。她的老花鏡瞬間閃現的光，當她抬起頭來，看看誰進來了。

打開的窗口房間裡放著一個大的灰色皮箱。雖然它是由明亮，薄荷綠牆和窗簾歡快地飄揚過它，這是個寂寞的景象給我。對於箱子，裡面會從家裡的唯一的東西，媽媽會跟她的時候，當她在一個星期的時間裡去往加拿大。

媽媽勉強獲得足夠為我們的家庭為衛生署的督察在一個小鎮在菲律賓南部。除了支付定期賬單和費用，她堅持送三個孩子去一所私立天主教學校。但媽媽是令人欽佩的努力工作。每一天，她忍受 90 分鐘的車程到辦公室，然後再返回到我們簡陋的家在達沃市。通常，她穿過岩石的道路前往遙遠的村莊做田野調查，有時回家曬黑和隱隱作痛。

我是 11 歲。媽媽決定在 2001 年去多倫多，在那裡她的弟弟住，把工作作為家庭傭工。我的哥哥約翰尼，姐姐茱莉比，我會留在我們的叔叔和嬸嬸。

我們三個都掰指頭數日子，不知道什麼時候我們會看到媽媽，她離開後再次。

「我只是希望她沒有去，」我說。

「她想走。她想擺脫你，」約翰尼會取笑我。「你是如此令人難以置信的臭，當你從學校趕回家，她無非是想轉移到其他國家。」

我會堅持我的舌頭在他，但我知道他在開玩笑。媽媽希望通過在國外工作，改善我們的生活。她想成為一個好的供應商，她肩負起了她的婚姻為我們的父親離開的時候我七歲。在那之後，我們再也沒有見過他，或從他那裡得到任何支持。

提高對自己的三個孩子非常困難。有時在考試，我的兄弟姐妹，我不得不忍受嚴重的修女之前，求他們讓我們參加考試，即使我們的學費沒繳清。

我經常想，為什麼媽媽沒有把我們送到一所公立學校。這本來倖免她三千

披索（$64）每月。但我確切知道，她會說，如果我曾經有膽量問她：「教育非常重要。我們可能不富有，但至少我已經給你了無價的禮物。」

媽媽把我拉回來，從我的想法，因為我盯著角落的旅行箱。抱著她的針線，她說：「英戴，請你為我做？」

我明白了她的意思。她總是問我把線穿過針孔，當她縫製。有時它讓我生氣。

「為什麼總是我？」我要求後，她就打斷了我，而我是打拋石子遊戲與我的表兄弟。

「因為你有更清晰的視力，」媽媽說。

「好了，阿茱有明顯的視力，」我說，指的是茱莉比。「你為什麼不叫她呢？」

「因為你最年輕，」她簡單地回答道。

今天，當我拿著針線的媽媽，我注意到，她的鼻子閃爍著汗水的努力工作。過了一段時間，我穿線，但我知道，如果我讓媽媽去做，她會錯過飛往加拿大之前，她不會成功。

我意識到在那一刻，穿線針是一個非常小的支持跟媽媽相比，現在她已經完成，並會做給我們。當我說完，我把針還她。我不敢看她的眼睛，因為我知道她會在我看到——悲傷。

這種感覺變更糟，因為在行李箱我的目光落在了一遍，然後將針在她的手。想也沒想，我說：「誰會在加拿大把線穿過針嗎？」

我想讓她知道，我會把每一個線程，通過在達沃市的每針只是為了讓她留下來，即使它確實煩擾我。

眼淚滾滾而下媽媽的臉頰。她伸出手，把我拉進一個緊密的擁抱。我開始哭了，因為在那一刻，我知道媽媽的答案是——沒有人。

我意識到，她需要從家裡的一切，隨身攜帶不在一個手提箱，但她的體內。據我所知，所有這些累人的日子媽媽花了登山穿越偏僻的村莊沒有什麼比

她還可以做。她會願意要經過千針洞，這是否意味著更好的生活。我知道在那一刻，即使媽媽是離我們很遠，我們的心將永遠被縫在一起。

NM1

#02 中國大陸加大反壟斷動作

當將近 100 個政府的反壟斷調查同時進軍四間在中國微軟的辦公室上個月，他們不是去找茶聊八卦的。

在微軟內部被定性為「突擊檢查」，從中國國家工商行政管理總局和商務部的代理人詢問公司的副總裁和其他高級管理人員，複製合同和下載大量的數據，包括電子郵件和其他內部溝通。

突擊在微軟上一舉站出來為它的規模，但它只是幾十個在中國各地類似的行動最近已掀起警報在世界各地的會議室其中的一個。中國監管機構似乎擴大執行反壟斷法，以及外國公司擔心，他們可能很容易成為目標，從機構和地方政府希望能夠在打動習近平，中共領導人誰推動了愛國的復甦和科技的官員的卓越願景。

外國公司擔心調查可能代表保護主義籠罩在監管公正性的崛起，但主要是為了促進中國企業，特別是強大的國有企業。政府說，它是利用反壟斷法，在 2008 年成立之初，用以保障消費者。

「如果中國將成為全球反壟斷制度的第三站，隨著美國和歐盟，而這顯然來了，那麼關鍵的問題是，什麼樣的做法是中國要走？」傅里斯比，以美國中國商務理事會在華盛頓的總裁說：「是不是會更社會主義的國營模式，或者可能是要更加以市場和消費者為導向的模式，或者兩者之間的東西？我不認為我們已經知道答案了。」

並非一切都在中國堆放起來反對跨國公司，其中有一些自己的政治盟友和合作夥伴。也不是在中國，微軟等公司都面臨著消費者的憤怒和監管審查的這不是唯一國家。但是，跨國公司似乎面臨著在中國新的重大挑戰。

「中國有非常龐大的官僚機構，但是每個機構都有其動機和任務，所以當他們執法，他們嘗試讓自己的利益最大化，」張湖月，在法律上，在倫敦大學國王學院的講師說。「但我不會低估了一些非常大的跨國公司的力量，因為這些

公司也非常雄厚的，並已在中國很好的聯繫。」對現在而言，雖然，微軟；聖地亞哥的芯片製造商高通；戴姆勒，德國汽車巨頭；和其他強大的企業都在防守。

最近幾個星期，調查人員一直特別忙——和公開露面——在他們的努力下，設置使得外國公司的辦公室突然搜查的格局。這些報告由當地媒體報導，由公司確認，其次是政府的聲明，該公司涉嫌違法定價，分銷和捆綁規則。

8 月 6 日，國家工商行政管理總局和商務部對中國兩個微軟辦公室進行跟進突擊，並在大連市搜索埃森哲的辦事處。在八月初，在國家發展和改革委員會官員搜查戴姆勒，它製造的賓士轎車的上海辦事處，並表示，他們計劃要罰款克萊斯勒和奧迪。 8 月 11 日，奧迪表示，在一個省，其中國合資公司的經銷商網絡打破了國家的反壟斷規則。一天後，通用汽車公司說，它已被聯絡來自中國當局。

這項改革委員會強制辦理定價侵犯中國的反壟斷法，並已研究如何汽車價格是由他們在中國的分銷商銷售的備品備件。對此，寶馬表示將降低配件價格。類似組件的降價最近已經由賓士和奧迪公布。

8 月 13 日，中國新聞媒體說的一個顧問，屬於一個政府委員會，已被解僱，被指控接受來自高通的支付，它正在調查有關違反反壟斷法的嫌疑。

該公司已製作了「大額支付」，以張昕竹，經濟學家，而他是一個反壟斷委員會的顧問，報告說。

在外企，很少會出現在中國比微軟有更好的連接。其聯合創始人比爾·蓋茲，曾與見面習近平幾次，他主持習先生的前任，胡錦濤主席在 2006 年晚宴，在他的家在麥迪納，華盛頓。

目前尚不清楚對微軟管理階層中國當局如何精確相信他們違反了國家的法律。中國官員曾公開表示，調查是相關與軟件的相容性，捆綁和文件核查問題的「視窗系統」和 Office 系列。

本月初，在人民日報，黨的主要報紙，告訴外國企業來適應更嚴格的審查：「每樣的企業應該調整自己的行為和思維，以這個新的法定正常。」

NM2

#03 海洋動物藏身於無掩蔽處

海洋，從而彌補了超過 90%的地球上的可以居住空間，是充滿了幾乎看不見的動物。

為了說明為什麼這樣，江森博士，生物學杜克大學在北卡羅萊納州的教授江森，開始了透過一個可怕的場景在他的會談。假設就在這時，一名持槍男子衝進房間，在觀眾射擊。當然，人們會爭奪搶背後的椅子和牆壁覆蓋。

他的觀點是：將會是有的地方，可以試圖隱瞞。

在陸地上，許多動物偽裝自己在之間一片葉子和地形環境；在沿海水域，海洋生物融入沙子或發現珊瑚或岩石中避難。但在大洋深處，生物漂浮在水面上沒有地方可以尋找避難。

透明度是最明顯的戰略，並且是一個江森博士首先開始了近 20 年前的研究。

透明度不只是缺乏色素沉著。白化病，江森博士指出，並不是看不見的；相反，整個身體必須吸收或散射少量的越好。

散射是一個挑戰。當光穿過成折射的不同的折射係數，這往往是成正比對於密度的材料，在光的一部分反射，而另一部分彎曲。這在很大程度上解釋了為什麼人們可以搜索長和寬的透明的牛或鴿子，並沒有找到一個：空氣的密度是如此比肉的少很多，即使是變成透明的陸生動物可能會很容易地從它的反射發現。

水是緻密得多，並且身體組織大致是像水的密度，大大減少散射的量。但有些器官比其它的更緻密，並且在透明的動物收拾內側方法不同，以最小化反射。

他從深處長大的透視生物，江森博士的測量結果發現，光的 20%至 90%通過，不受干擾。「你可以通過這些動物讀一本書，」他說。

但透明性可以使變得複雜生命，並且在表面附近的透明的生物可能曬黑，

不僅對皮膚但裡面得。為了保護自己免於受到紫外線，「這些人基本上都有防曬霜在自己的透明組織裡面，」江森博士說。

進化已經想出了兩個其他形式的隱身技術：鏡子和生物燈泡。

一些大鱺通過尋找輪廓上面找到自己的食物。「你看到許多動物仰視的眼睛，甚至有一個大眼睛仰視和『正常』的眼睛看向一邊魷魚，」說：哈多克，科學家在蒙特利灣水族館研究所的莫斯碼頭，加利福尼亞。

魚類銀色邊如鯡魚和沙丁魚的是鏡子的系統：它們反映了下降流光線，很多是那方式的天空的一部分，有時用玻璃摩天大樓反映。因此，從下面捕食者會看到藍色的水，不是魚，上面的。

埃里克·丹頓，英國海洋生物學家，20 世紀 60 年代的研究鏡像魚，想通了，鏡子是垂直的，將最大化的那錯覺。

第三個戰略，稱之為反照明，還試圖模仿下降流的光。但是，而不是鏡子，動物產生自己的光芒，就像螢火蟲做被譽為發光的器官。

採用反照明的動物，確保它們產生的光指朝向下。

「他們不希望光漏到一邊，讓他們做為脆弱的，所以他們有透鏡，反射鏡和濾光片在上他們發光器官，」哈多克博士說。

有些動物已經進化的方式來打敗偽裝。魷魚和蝦的品種有眼睛，可以光的偏振，這是很多昆蟲可以做，但人們不能沒有偏光太陽鏡來做區分。

光子——光的粒子——可以被認為是有尾鰭的箭頭表示振盪磁場和電場，並且偏振代表場傳播的方向。對於人眼，反射光的顏色不變。當反射，極化角的變化。

在天空中，濾光極化向下到深度變化的當太陽移動，對於眼那可以告訴偏振之間的差異，鏡像魚突然伸出。

「事實證明，雖然偽裝才是真的好，你真的是可以利用偏振視覺打破它，」江森博士說。

這可能，例如，允許魷魚當場接近的餓金槍魚而逃。

「我們只是被包圍有一個完全神秘的世界，」江森博士說。「我們不能看到它實際上意味著我們忽略它的大部分時間。」

NM3

<u>#05 把碳封存在地下的方法</u>

加拿大薩斯克徹溫省伊斯得芬，一個新的燃煤發電廠建起在這裡廣闊的大草原，以取代一個發散出很多煙灰，衣服在禁區外乾燥將覆蓋砂礫這麼多的。

但與即使是最現代化的燃煤電廠，工廠的煙囪還在排放二氧化碳，看不見的氣體，是主要的貢獻者，對全球變暖的數。所以今年秋天，管道和儲罐的一個新的迷宮會吸取 90%的二氧化碳來自鍋爐，因此可以被運出埋藏，地下深處。

這種努力將是它的種類在發電廠的第一個重大的，25 萬輛汽車的等價物。並且，至少理論上，該二氧化碳將被隔離從大氣層永遠。

全世界，煤炭消費量在 2020 年將有約兩倍它是在 2000 年，根據美國能源情報署，並會繼續增長幾十年。

「若你想繼續使用這些化石類，這意味著清理自己的排放量，」愛丁堡大學的哈塞爾丁說。碳捕獲，他說，「這樣做的一個最好的辦法。」

然而，因為它需要那麼多精力，吸乾碳減少電廠的能力，使電力。有否二氧化碳可以安全儲存在地下的基本問題。並且該技術昂貴。僅更新薩斯克徹溫省電廠收 12 億。

在密西西比州中部的松樹林，另一個碳捕獲的努力正在形成，在一個巨大的新發電廠將被送入一個穩定的煤炭供應從露天礦隔壁。

哈靈頓，業務經理，說工人的軍隊在切割，焊接和檢測地下。工廠老闆，南方公司，希望能打開建設明年。但它比薩斯克徹溫省的努力更加複雜，價格標籤是 55 億。

美國和其他國家幫助一些項目——加拿大給 2.2 億美元到薩斯克徹溫省工廠的老闆，薩省電力，和南方公司收到 2.7 億美元從能源的美國能源部——但成本足夠高，其他少數電力公司有做超出研究的概念。

該技術已經存在近一個世紀，一些煉油廠和其他工廠，包括在伊利諾伊

州，北達科他州，加拿大和挪威使用。

但是從釋放在發電廠的氣體的漩渦中除去二氧化碳是具有挑戰性。設備是巨大的。在薩斯克徹溫省的廠，被稱為邊界大壩，液體化學品被噴灑到燃燒氣體後鎖上二氧化碳分子。再脫掉，其中二氧化碳終於拉開，高近 50 米。

此外，效率損失，因為一些通常會產生電力的蒸汽進入脫除器來代替。

和一個可怕的馬達壓縮二氧化碳——直到它實際上變成液體——運輸。總而言之，二氧化碳的捕獲在邊界大壩將削弱發電約 20%。液體注入地下深處可能存在的問題，也。從石油和天然氣生產廢水抽入地面已被關連到在美國的小地震。

二氧化碳可能玷污飲用水，或氣泡向上進入大氣，擊敗整個目的。

儘管如此，二氧化碳已被掩埋在世界各地的一些少的問題。在挪威，大約一百萬公噸已存儲每年自 1996 年以來，注入砂岩之下北海約 900 米。大部分邊界大壩的二氧化碳將成為一種工具來提取和使用的油。

正在通過 60 公里管道至油田出售和出廠後，二氧化碳將被泵入老井，其中，將與在內部的油混合，從而使其更好地流動。該過程被稱為強化油採收。在石油和天然氣行業已經幾十年來這樣做，主要是與天然存在的二氧化碳積聚在地下。不過，每年在北美，二氧化碳行業超過 13 億噸被使用。

這種做法可能在許多油田擴大，專家說，可能存儲數十億的二氧化碳噸，服務為橋樑，以一天當它成為必要的，經濟的，存放在其他地方的氣體。

預後為世界各地的碳捕獲是不清楚的。

如果美國前進與總統歐巴馬的計劃，減少碳排放，中國和其他國家可能也會做出更大步伐。

「這將如何發揮出隨著時間的推移，很難說，」卡內基美隆大學在賓州的魯本說：「不可避免地，會有技術能力，成本和政治現實之間的平衡。」

NM4

#07 鐵路改運石油 不利農民

在北達科他州的能源勘探憤怒的步伐創造了農民的糧食的出貨量已連續被積壓了，由石油通過鐵路一個龐大的新運送的危機，導致數百萬美元的農業損失和生產速度較慢的早餐穀物巨頭通用磨坊。

積壓只會變得更糟，農民說，正當他們最近準備對什麼是預計小麥和大豆的創紀錄收成。

「如果我們不能得到這個東西出來很快，它的很多只是會去在地上腐爛，」赫傑爾，誰長大大豆和小麥在一個小鎮附近在這裡說。

雖然在北達科他州的能源熱潮，導致了 2.8%的失業率，最低的在美國，不足之處還為農民誰一直是國家經濟的支柱是困難時期。

農業是北達科他州的一號產業幾十年，代表上有四分之一的經濟基礎，但最近的統計數據顯示，石油和天然氣已成為最大的貢獻者對於國家的國內生產總值。

鐵路一直是北達科他州的交通運輸系統的骨幹和最可靠的方法，為農民運動作物——在奧勒岡州波特蘭市的端口；西雅圖；和溫哥華，從該散裝穀物的跨太平洋運到亞洲；和東海岸港口如紐約州奧爾巴尼，從它被運到歐洲。

但報告提交給政府表明，截至 8 月 22 日的一周中，伯靈頓北方聖大非鐵路（BNSF）——北達科他州最大的鐵路——有 1336 軌道車等運送糧食和其他產品的積壓。另一位鐵路公路，加拿大太平洋，有近 1000 輛積壓。

對於農民來說，拖延往往意味著食品巨頭不能等待數週或數月，因為他們需要做出麥片，麵包等產品陣列中糧取消訂單。

美國農業部官員近日表示，他們特別關注的是加拿大太平洋將無法在十月前完成近三萬個要求任務，從農民和其他人的軌道車。

「這條鐵路的積壓是一個全國性的問題，」北達科他州的參議員海特坎普說。「農民無法獲得這些穀物市場不僅是農業問題，但對於產生麥片，麵包等商

品的公司。」

由北達科他州立大學海特坎普女士的要求進行的一項最新研究發現，軌道交通擠塞的費用可以在國營農場者超過 1.6 億美元，因為在當地的糧食供應已經降低了價格。

這項研究還發現，農民將失去 6700 萬美元的小麥，玉米和大豆對於收入從一月到四月中旬。各地 9500 萬美元或者有更多的損失預計，如果農民不能移動他們的剩餘農作物。

食品公司說，他們都感受到了延遲出貨的影響。通用磨坊食品，麥片的明尼蘇達州的製造商，「歡樂穀」穀物的大眾品牌，告訴投資者在三月份，它已經失去了生產 62 天——多達 4%的出放——在二月份結束的季度，因為冬天的物流問題，包括鐵路，汽車擁堵。

在 8 月的財報，嘉吉公司，其他一個位於明尼蘇達州的食品業巨頭，報淨收益，這部分歸因於降「涉及鐵路車短青睞變高的成本。」

農民和農業組織說，鐵路運營商顯然有利於中石油更豐厚的運輸。

原油在北達科他州的鐵路貨運自 2008 年以來已飆升，而國家目前每天生產約萬桶。約 60%的石油搭乘火車從國家到遙遠的煉油西部巴肯油田。有幾個管線出貨。

BNSF 與加拿大太平洋維持他們的石油運輸沒有替代作物的出貨量。

「當然，我們正在出貨，這些天最大的不同是油，」BNSF 的羅斯說「但我們不會偏袒一種類型的產品比另一種。」

儘管如此，BNSF 在可行權在北達科他州約四億美元，興建更多的軌道，聘請新員工，並添加軌道車。羅斯先生說，「我們正在做這項投資在我們的基礎設施，以確保我們把事情恢復正常。」
